# Effect of high temperatures on sex ratio and differential expression analysis (RNA-seq) of sex-determining genes in *Oreochromis niloticus* from different river basins in Benin

**DOI:** 10.1093/eep/dvad009

**Published:** 2024-01-13

**Authors:** Mohammed Nambyl A Fagbémi, Renaud Nivelle, Marc Muller, Charles Mélard, Philippe Lalèyè, Carole Rougeot

**Affiliations:** Aquaculture Research and Education Centre (CEFRA), Liège University, query author on which is prefered, 10 Chemin de la Justice B-4500, Tihange, Belgium; Laboratory of Hydrobiology and Aquaculture (LHA), Faculty of Agricultural Sciences, University of Abomey-Calavi, 01 BP: 526, Cotonou, Benin; Aquaculture Research and Education Centre (CEFRA), Liège University, query author on which is prefered, 10 Chemin de la Justice B-4500, Tihange, Belgium; Laboratory for Organogenesis and Regeneration (LOR), Interdisciplinary Research Institute in Biomedical Sciences (GIGA-I3), Liège University, Sart Tilman, Liège, Belgium; Laboratory for Organogenesis and Regeneration (LOR), Interdisciplinary Research Institute in Biomedical Sciences (GIGA-I3), Liège University, Sart Tilman, Liège, Belgium; Aquaculture Research and Education Centre (CEFRA), Liège University, query author on which is prefered, 10 Chemin de la Justice B-4500, Tihange, Belgium; Laboratory of Hydrobiology and Aquaculture (LHA), Faculty of Agricultural Sciences, University of Abomey-Calavi, 01 BP: 526, Cotonou, Benin; Aquaculture Research and Education Centre (CEFRA), Liège University, query author on which is prefered, 10 Chemin de la Justice B-4500, Tihange, Belgium

**Keywords:** *Oreochromis niloticus*, sex determination, gene expression, temperature, sex ratio, RNA-seq

## Abstract

The high temperature sex reversal process leading to functional phenotypic masculinization during development has been widely described in Nile tilapia (*Oreochromis n iloticus*) under laboratory or aquaculture conditions and in the wild. In this study, we selected five wild populations of *O. niloticus* from different river basins in Benin and produced twenty full-sib families of mixed-sex (XY and XX) by natural reproduction. Progenies were exposed to room temperature or high (36.5°C) temperatures between 10 and 30 days post-fertilization (dpf). In control groups, we observed sex ratios from 40% to 60% males as expected, except for 3 families from the Gobé region which showed a bias towards males. High temperature treatment significantly increased male rates in each family up to 88%. Transcriptome analysis was performed by RNA-sequencing (RNA-seq) on brains and gonads from control and treated batches of six families at 15 dpf and 40 dpf. Analysis of differentially expressed genes, differentially spliced genes, and correlations with sex reversal was performed. In 40 dpf gonads, genes involved in sex determination such as *dmrt1, cyp11c1, amh, cyp19a1b, ara*, and *dax1* were upregulated. In 15 dpf brains, a negative correlation was found between the expression of *cyp19a1b* and the reversal rate, while at 40 dpf a negative correlation was found between the expression of *foxl2, cyp11c1*, and *sf1* and positive correlation was found between *dmrt1* expression and reversal rate. Ontology analysis of the genes affected by high temperatures revealed that male sex differentiation processes, primary male sexual characteristics, autophagy, and cilium organization were affected. Based on these results, we conclude that sex reversal by high temperature treatment leads to similar modifications of the transcriptomes in the gonads and brains in offspring of different natural populations of Nile tilapia, which thus may activate a common cascade of reactions inducing sex reversal in progenies.

**Key Messages**

- Our RNA-seq data revealed a complex of genes (*kmt5c, aebp2, abhd4*, and *jarid2*) that could have important roles in sex differentiation and in the resistance of some females to the effects of high temperatures through the phenomenon of apoptosis

## Abbreviations

↑Exp: significant upregulated expression,

→Exp: significant downregulated expression,

+Corr: significant positive correlation,

–Corr: significant negative correlation,

↑exp: no significant upregulated expression,

→exp: no significant downregulated expression.

## Introduction

In developmental biology, the lack of a conserved and generalized mechanism to determine sex is a major paradox [1]. Sex determination can be defined as the genetic and/or environmental processes that influence the definition of sex [2, 3]. With more than 35 704 existent species [4], fish exhibit a wide variety of sex determination mechanisms [5] making them an excellent model to study the evolution of the sex determination process [2]. In mammals and birds, sex is genetically driven by a chromosomal sex determination system (CSD), with male heterogamy (XY) in mammals and female heterogamy (ZW) in birds. This process is under the control of the major *sry* gene in mammals and the *dmrt1* gene in birds [1].

Sex determination in fish can be very plastic, including genetic and environmental influences [2]. Fish species may exhibit CSD, polygenic sex determination (PSD) with the combined action of several pro-male and pro-female (in favour of male and female) genetic factors, and sex determination only dependent on temperature (TSD) (Penman and Piferrer, 2008). Nile tilapia is a gonochoristic teleost with an XX/XY sex determination system [6]. Its phenotypic sex can be established by a major determinant on linkage group 1 [7–9], the interaction of minor autosomal factors [10, 11], and environmental factors such as high temperature [12–15]. Temperatures up to 34°C may efficiently masculinize progenies if they are applied approximately 10 days post fertilization (dpf) for at least 10 days [12–14, 16]. This period of sexual lability can last from weeks to years depending on the species [17, 18], opening a wide developmental window in which the sexual phenotype can be influenced by abiotic or biotic factors [6, 19]. Since the first evidence of TSD in fish in Atlantic silverside (*Menidia menidia*) in 1981 [20], almost 60 species displaying TSD or genetic sex determination with a temperature effect TE (Genotypic Sex Determination + TE) have been described [21].

The effect of high temperatures on sex phenotype reversal undoubtedly occurs through a disruption of the normal genetic process for sex determination and differentiation. Thus, the effects of temperature on gene expression during sex differentiation was investigated in different teleost species, such as the African catfish (*Clarias gariepinus*) [22, 23], European sea bass (*Dicentrarchus labrax*) [24, 25], Japanese flounder (*Paralichthys olivaceus*) [26], pejerrey (*Odontesthes bonariensis*) [27], and Nile tilapia [28, 29]. These studies have shown that high temperature affects the sexual differentiation cascade, acting directly on the ovarian differentiation pathway and activating testis development [30]. Many studies have thus shown that high-temperature treatment affects mRNA expression of genes such as *dmrt1, amh, sox9a, cyp19a1, foxl2, wt1, sf1*, and *hsps* [28, 29, 31, 32]. It also appears from different studies that several key genes like *dmrt1, amh, sox9* [33, 34], *cyp19a1a*, or *foxl2* [35] are determinants of sex differentiation. A gene such as *dmrt1*, for example, is a functionally conserved factor that promotes testicular differentiation across vertebrates and loss of *dmrt1* upregulates *foxl2* and *cyp19a1a* to induce ovarian development. Thus, *dmrt1* plays a crucial role in vertebrate sex determination/differentiation by antagonizing *foxl2* [36–38].

While masculinization of Nile tilapia populations is widely used in aquaculture (by hormone exposure), phenotypic XX males were also reported in the wild [10, 39]. This observation raises the question of whether TSD plays a role in the adaptation of wild populations to specific environmental thermal conditions. One approach to answer this question would be to compare populations from different locations with regard to their capacity/propensity to respond to temperature-induced sex reversal. Recently, we compared the growth and breeding [40] characteristics of Nile tilapia captured in different locations in Benin and we determined their genetic relationships [41]. The present work aims to study: (i) the effect of geographical origin on high-temperature sex reversal by investigating the sex ratios of populations of *O. niloticus* from 5 different locations in Benin, and (ii) the effects of high-temperature treatment on the transcriptome through the study of differential expression of genes involved in the sex determination and differentiation processes in fish.

## Results

### Effect of High Temperature on Survival and Male Rates

#### Effect of High Temperature on Survival

Among the 20 tested progenies, survival rates at the end of the treatment period (31 dpf) ranged from 54% to 99% in the control batches and from 54% to 96% in treated batches, *e.g.* for Gbassa and Nangbéto, respectively (Supplementary Fig. S1A). There is no significant difference (*P* > 0.05) in survival rates between controls and treated batches of families GB_F3, GO_F1, GO_F4, NGT_F1, NGT_F3, SH_F1, and SH_F2, while families GB_F1, NGT_F4, and SH_F3, in contrast, revealed a significantly higher survival after high temperature treatment (from 81%, 81%, and 76%, respectively, to 95%, 92%, and 85.3%) (Supplementary Table S1).

**Figure 1:**
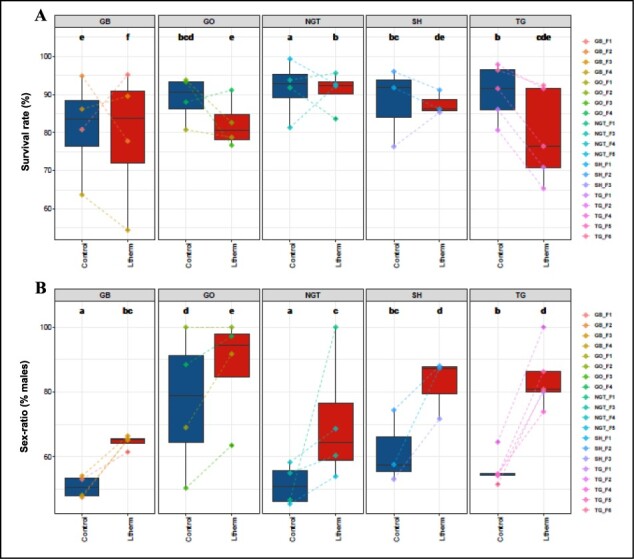
Survival rates (A) and sex ratios (B) in control (blue) and high temperature treated (red) batches for each population. Median values are indicated by a vertical line, the boxes encompass the 25% and 75% quartiles, the dots represent the values for each individual batch, while the lines joining two dots link the corresponding control and treated batches of the same family. GB: Gbassa, NGT: Nangbéto, SH: Sohoumè, TG: Togbadji

At the population level in the control batches, mean survival rates ranged from 84 ± 13% to 92 ± 8% and median values ranged from 84% to 93% for Gbassa and Nangbéto, respectively. In the treated batches, mean survival rates ranged from 79 ± 12% to 91 ± 5% and median values ranged from 76% to 92% for Togbadji and Nangbéto, respectively (Supplementary Table S2 and Fig. 1A). Overall, high temperature treatment significantly reduced the survival rate of fry in the treated batches compared to control batches (Fig. 1A). The inter-population comparison reveals that the Nangbéto population displayed a better survival rate (91 ± 5% and 92%) in the treated batches compared to the other populations and therefore a greater resistance to high temperature (Supplementary Table S2).

#### Effect of High Temperatures on the Sex Ratio

The sex ratio in each batch was determined at 90 dpf. Within families, the male ratio ranged from 45.5% to 100% for control batches and from 54% to 100% for treated batches (Supplementary Fig. S1B). Most of the control batches displayed a balanced, theoretically expected 50:50 sex ratio, with the exception of three out of four families in the Gobé population (GO_F1, GO_F2, and GO_F4) and one Sohoumè family (SH_F2) which displayed significantly (*P* < 0.001) skewed ratios towards males (Supplementary Table S1 and Supplementary Fig. S1B). After high temperature treatment, 11 out of 20 families displayed a significantly increased male ratio relative to their respective controls, with the Togbadji population recording a significant male bias within four out of five families (Supplementary Fig. S1B). On a population level, the male ratio ranged from 51 ± 3% to 77 ± 22% in the control batches and from 65 ± 2% to 88 ± 17% in the treated batches, for Gbassa and Gobé, respectively (Table 1). All populations experienced a significant increase in the mean male ratio in the treated batches compared to the control batches, with the treated Gobé population displaying the highest male ratio (a mean of 88 ± 17% and a median of 95%) compared to the other populations (Table 1, Fig. 1B). The Togbadji, Gobé, and Sohoumè populations displayed the highest mean reversal rates (IRs) of 66 ± 22%, 58 ± 28%, and 54 ± 15%, respectively (Table 1). At the all-populations level, the increase in the percentage of males in the high-temperature treated batches was significant compared to the control batches (from 65 ± 2% to 88 ± 17%).

**Table 1:** Means and median values of male rates in each population

		Control				Ltherm				
Populations	N family	N sexed	Mean male ratio ± SD (%)	Median male ratio [P25−P75]	Group	N sexed	Mean male ratio ± SD (%)	Median male ratio [P25−P75]	Group	Mean inversion rates (IR)
Gbassa	4	574	51 ± 3	51 [48–53]	a	686	65 ± 2	65 [64–66]	bc	28 ± 7
Gobé	4	715	77 ± 22	79 [64–91]	d	691	88 ± 17	95 [85–98]	e	58 ± 28
Nangbéto	4	387	51 ± 6	51 [46–56]	a	451	71 ± 20	65 [59–77]	c	38 ± 42
Sohoumè	3	238	62 ± 11	58 [55–66]	bc	299	82 ± 9	87 [80–88]	d	54 ± 15
Togbadji	5	672	56 ± 5	54.4 [54–55]	b	684	84 ± 10	81 [80–86]	d	66 ± 22

In a row, treatments with different letters are significantly different (*P* < 0.05) between treatments (Control/Ltherm). In a column for the same treatment under consideration, populations with different letters are significantly different (*P* < 0.05). N family: number of tested families, N sexed: number of sexed fish, Group: results of statistical tests of significance between control and treated batches.

To further investigate the thermosensibility of these different populations, we analysed whether we could find a correlation between survival and reversal rates. Overall, a negative correlation (−0.23) was observed between the IR and the relative survival rate (RSR) (Table 2). Individual populations revealed a more significant negative correlation; only the Gobé population showed a positive correlation (0.80) between these two parameters (Table 2).

**Table 2:** Correlation between RSR and IR

	GB_IR	GO_IR	NGT_ IR	SH_ IR	TG_ IR	All_IR
GB_RSR/31 dpf	−1					
GO_RSR/31 dpf		0.8				
NGT_ RSR/31 dpf			−0.4			
SH_ RSR/31 dpf				−1		
TG_ RSR/31 dpf					−0.5	
All_ RSR/31 dpf						−0.23

Furthermore, we tested for correlations between male ratios in control and treated batches and their corresponding survival rates. A significant positive correlation (0.58) was observed between the percentage of males in the control batches and the male ratio in the corresponding treated ones, as was expected. A positive correlation (0.44) between the survival rate at 31 dpf in the control batches and the survival rate at 31 dpf in the treated batch was also observed (Supplementary Table S3).

### Effects of High Temperature Treatments on Gonad and Brain Transcriptomes at 15 Dpf and 40 Dpf

#### Characteristics of RNA-Seq Data

To identify genes’ whose expression affected by the high temperature treatment and to investigate the different signalling pathways involved in sex reversal, we performed RNA-Seq analysis. Samples were taken from control and treated batches at 15 dpf, in the middle of the high temperature treatment period, and at 40 dpf, ten days after the end of the treatment. The larvae were dissected to obtain gonads and brains. RNA extraction was performed and the RNA of 48 samples (24 at 15 dpf and 24 at 40 dpf for gonads and brains, respectively) was collected representing six families with very different reversal rates (GB_F1, IR = 18; GB_F2, IR = 34; GO_F1, IR = 73; GO_F3, IR = 26; NGT_F1, IR = 100; and NGT_F3, IR = 12). For all samples, a number of reads ranging from 43 to 48 million (Supplementary Table S4) was generated. The RNA integrity Number score values of the extracted RNAs ranged from 6 to 9 while the TIN score values ranged from 73 ± 3 to 87 ± 1 (Supplementary Table S4 and Supplementary Fig. S2A) showing that the quality of our extracted RNA and the sequencing was good. Good read coverage density was also achieved through the Gen Body Coverage graphs (Supplementary Fig. 2B). From the generated reads, the mean single mapping rates on the tilapia *O. niloticus* genome ranged from 81 ± 2% to 90 ± 1% for the 48 sequenced samples (Supplementary Table S4). All these parameters indicate a high integrity of the RNAs used.

**Figure 2:**
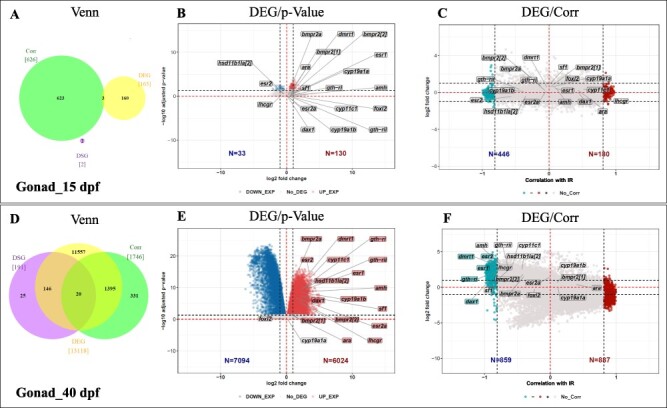
Comparison of gene expression in gonads of high-temperature-treated juveniles to that of controls (Ltherm versus control) at 15 dpf (A–C) and 40 dpf (D–F). For each stage, the data are presented as a Venn diagram, a volcano plot, and a corr-plot. (A, D) Venn diagrams representing the number of differentially expressed genes (yellow), differentially spliced genes (purple), and Corrs (green), respectively, as well as the number of genes common to the different categories. (B, E) Volcano plot representing the log2-fold change in gene expression versus statistical significance (−log10 of adjusted *P*-value). Coloured dots represent the significantly regulated genes (*P*-value < 0.05), red for upregulated, blue for downregulated. (C, F) Plot representing the log2-fold induction versus the correlation coefficient of this log2-fold change with the reversal rate (IR) displayed by the different families. Significant correlation is shown by the coloured dots, positively correlated in red, negatively in blue. Rectangles contain the names of genes that were selected from the literature as being relevant to sexual differentiation and maturation; the colour of the rectangle indicates significance, either for differential expression or correlation with IR

#### Differentially Expressed Genes, Differentially Spliced Genes, and IR Correlated Genes

We used the collected RNA-Seq data to identify the genes that were differentially expressed genes (DEGs) or differentially spliced genes (DSGs) during and after high temperature treatment in the families selected for transcriptomic analysis. Furthermore, we took advantage of the fact that the samples that were sequenced belonged to different families with various reversal rates to identify genes with expression levels that were positively or negatively correlated (±Corrs) with the observed IRs, assuming that such genes were more likely to be involved in the sex reversal process.

In 15 dpf gonads, 791 genes were identified with 163 DEGs, 2 DSGs, and 626 Corrs, while only 3 were common to DEG/Corr (Fig. 2A). In 40 dpf gonads, 15 055 genes were found, with 13 118 DEGs, 191 DSGs, and 1746 Corrs, while overlaps were observed in the gene list for 146 genes in DEG/DSG, 1395 in DEG/Corrs, and 20 in all three lists (Fig. 2D).

We first compared these gene lists to a list of genes found in the literature and considered to be associated with sexual differentiation or maturation (Supplementary Table S5). Interestingly, none of genes affected by the treatment in 15 dpf gonads was in this list (Fig. 2B, C), probably indicating that the process of sexual differentiation has not started at this stage. In contrast, in 40 dpf gonads, there was a significant upregulation of the genes of interest *dmrt1, esr2, cyp11c1, gth-ri, gth-rii, hsd11b1Ia(2), esr1, amh, dax1, cyp19a1b, ara, cyp21a2, ihcgr, bmpr2(1), bmpr2(2)*, and *sf1* (Fig. 2E). A negative correlation of the expression of *dmrt1, esr1, esr2, gth-ri*, and *dax1* genes with IR was also observed at this stage (Fig. 2F).

**Figure 3:**
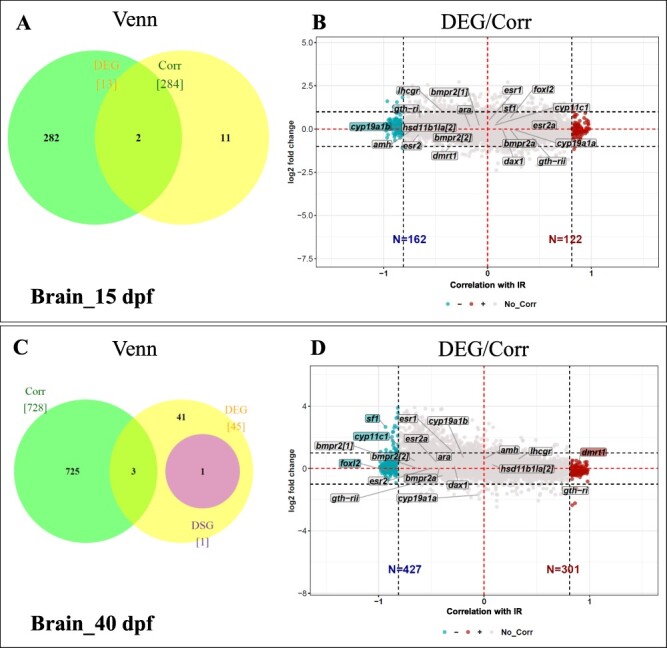
Comparison of gene expression in brains of high-temperature-treated juveniles to that of controls (Ltherm versus control) at 15 dpf (A–B) and 40 dpf (C–D). For each stage, the data are presented as a Venn diagram (A, C) and a Corr-plots (B, D). Venn diagrams representing the number of differentially expressed genes (yellow), differentially spliced genes (purple), and Corrs (green), respectively, as well as the number of genes common to the different categories. (B, D) Plot representing the log2-fold induction versus the correlation coefficient of this log2-fold change with the reversal rate (IR) displayed by the different families. Significant correlation is shown by the coloured dots, positively correlated in red, negatively in blue. Rectangles contain the names of genes that were selected from the literature as being relevant to sexual differentiation and maturation; the colour of the rectangle indicates significance for correlation with IR

In 15 dpf brains, the expression of 297 genes was affected, with 13 DEGs, 284 Corrs, and 2 DEGs/Corrs (Fig. 3A), while at 40 dpf, 774 genes were identified, with 45 DEGs, 1 DSG, 728 Corrs, 3 DEG/Corr, and 1 DEG/DSG) (Fig. 3C). None of the DEGs or DSGs was present on our list of genes associated with sex differentiation or maturation; only a negative correlation was observed between the expression of the *cyp19a1b* gene for brain aromatase and IR at 15 dpf (Fig. 3B), while at 40 dpf, there was a positive correlation between the expression level of *dmrt1* and IR and a negative correlation between the expression levels of the *foxl2, cyp11c1*, and *sf1* genes and IR (Fig. 3D) (it should be noted that in the Ensembl database, the gene coding for the Foxl2 protein is no longer present even though its code ENSONIG00000020788 is still present and has been changed from a gene biotype to a pseudogene biotype. In contrast, *foxl2* is still present in the NCBI database, which still associates it with the same ENSONIG00000020788 code present in the Ensembl database).

Filtering the raw lists of identified genes in the gonads and brains at 15 dpf and in the brains at 40 dpf on the basis of both differentially expressed (up- or downregulated) and correlated with IR (+ or −) genes produced a list of 1 novel and 7 known genes. In 15 dpf gonads, the genes *abhd4* (↓Exp and −Corr), *osbpl3b* (↓Exp and −Corr), and *kmt5c* (↑Exp and +Corr); in 15 dpf brains, the genes *aebp2* (↓Exp and +Corr) and *nrf1* (↓Exp and +Corr); and in 40 dpf brains, the genes *opn1lw1* (↑Exp and −Corr), a novel gene (ENSONIG00000040860) (↑Exp and −Corr), and *mag* (↑Exp and −Corr) (Supplementary Table S6) were identified as genes that may play a key role in sex differentiation in tilapia in view of their different expression profiles and their correlation with IR. In addition, 11 genes emerged from the 15 dpf brains as being significantly regulated (↑↓Exp) but did not correlate (No_Corr) with IR (Supplementary Table S6).

For the 40 dpf gonads, following the same filtering process (DEG and Corr genes), a list of 75 genes (↑↓Exp and Corr) was identified. Within this gene list, in the gonads at 40 dpf, the five most significantly regulated (based on adjusted *P*-value) genes were *cyp24a1, sc5d(2), hmgb1b, LOC109196533*, and *pa2g4b* (Supplementary Table S7).

#### Gene Co-expression Network Analysis

**Figure 4:**
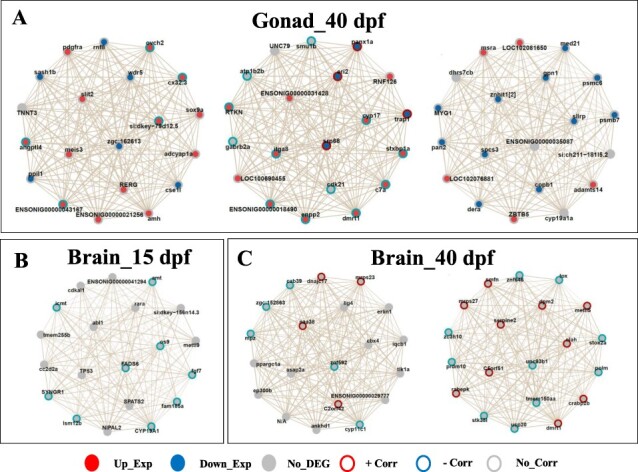
Networks of genes co-expressed with the *amh, cyp11c1, cyp19a1a, dmrt1, hsd11b1la*, and *hsd11b2* genes in the 40 dpf gonads (A), with the *cyp19a1b* gene in the 15 dpf brains (B) and with the *cyp11c1* and *dmrt1* genes in the 40 dpf brains (C). Genes with red centres denote upregulated expression and blue centres denote downregulated expression. The colour of the edges of the gene indicates a correlation of the gene expression with inversion rate (IR), red for a positive correlation and blue for a negative correlation. When a gene has no colouration then it is neither a differentially expressed gene (DEG) nor is its expression correlated to the IR (Corr) in our results but belongs to the network of genes co-expressed with our gene of interest. The thickness of the lines denotes the intensity of the links between the different genes of the network

Co-expression networks were obtained, taking into account all the genes that emerged from the RNA-seq analysis, using the fold-change values (per family) obtained after count per million (CPM) normalization of read count data to detect an expression correlation between the different genes. Co-regulation in opposite directions (up or down) was expressly allowed. For each of the 10 genes of interest, namely *amh, cyp11c1, cyp19a1a, cyp19a1b, dmrt1, hsd11b1la, hsd11b1lac(2), dax1 (nr0b1), sf1 (nr5a1)*, and *hsd11b2*, we constructed a co-expression network with the 19 genes (as shown in Fig. 4) whose expression correlated most at different stages and tissue types. DEG and Corr data for each gene were integrated into the graphical representations of the networks (Fig. 4). Interestingly, genes within a specific co-expression network were not always differentially expressed upon heat treatment (not DEGs), nor did their expression always correlate with IR (no-Corr). This observation underscores the need to analyse this type of effect using different methods.

The most interesting co-expression networks were found in the 40 dpf gonads emerging, respectively, from the *amh, dmrt1*, and *cyp19a1a* genes (Fig. 4A). In the 15 dpf brains, the most interesting network was the co-expression around the *cyp19a1b* gene (Fig. 4B), while in the 40 dpf brains it was those around the *cyp11c1* and *dmrt1* genes (Fig. 4C). The co-expressed gene networks obtained in the gonads at 40 dpf were predominantly composed of DEG genes, while those found at 15 and 40 dpf in the brains were composed of genes for which expressions were correlated with IR. In the 40 dpf gonads, genes such as *sox9a* (↑Exp, no-Corr), *adcyap1a* (↑Exp, no-Corr), *rerg* (↑Exp, no-Corr), and *cse1l* (↓Exp, no-Corr) were co-expressed with the *amh* gene; while for the *dmrt1* gene, we observed, for example, *c7a* (↑Exp, −Corr), *enpp2* (↑Exp, −Corr), *cdk21* (no-DEG, −Corr), and *itga8* (↑Exp, −Corr) which were co-expressed with *dmrt1* (Fig. 4A). The co-expression network of gonadal aromatase (*cyp19a1a)* stands out with co-expressed genes such as *adamts14* (↑Exp, no-Corr), *zbtb5* (↑Exp, no-Corr), *copb1* (↓Exp, no-Corr), *dera* (↓Exp, no-Corr), and *spcs3* (↓Exp, no-Corr).

**Figure 5:**
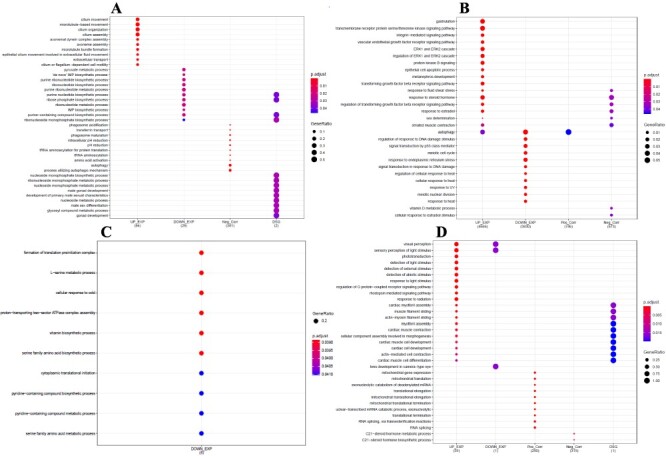
List of the most significant Gene Ontology (GO) terms and function of DEGs, Corrs, and DSGs associated with the effect of high temperatures in 15 dpf (A) and 40 dpf (B) gonads and in 15 dpf (C) and 40 dpf brains (D)

In 15 dpf brains, only the brain aromatase (*cyp19a1b*) co-expression network stands out with co-expressed genes such as: *fam185a* (−Corr), *fgf7* (−Corr), *syngr1* (−Corr), *fads6* (−Corr), *lsm12b* (−Corr), *os9* (−Corr), *amt* (−Corr), and *icmt* (−Corr) (Fig. 5B). In the 40 dpf brains, we observed the genes *c2orf42* (+Corr), *znf592* (−Corr), *naa38* (+Corr), *mrps23* (+Corr), and *cab39* (−Corr) that showed co-expression with the gene *cyp11c1*, while *dmrt1* remained co-expressed with genes such as *usp20* (−Corr), *crabp2b* (+Corr), *tmem150aa* (−Corr), *polm* (−Corr), and *stk38l* (−Corr) (Fig. 4C). All these genes, when co-expressed with the genes of interest, would interact with the latter at different stages and tissues to play a role in sex determination and differentiation in Nile tilapia. Table 3 lists the genes co-expressed with the 10 genes of interest in the most interesting co-expression networks obtained.

**Table 3:** List of genes co-expressed in co-expression networks with genes of interest identified in gonads and brains at 15 and 40 dpf

		Brain	
Gene of interest	Gonad 40 dpf	15 dpf	40 dpf
*cyp19a1a*	*adamts14, psmb7, psmc6, med21, LOC102081650, msra, dhrs7cb, myg1, pan2, LOC102076881, dera, zbtb5, copb1, spcs3, znhit1(2), gpn1, slirp, ENSONIG00000035087,*		
*cyp19a1b (CYP19A1)*		*fam185a, fgf7, mettl9, amt, icmt, syngr1, ism12b, fads6, os9*	
*cyp11c1*	*cmtm4, tnfrsf11b, scrib, snw1, lipea, clstn3, sf3b3, mob1bb, tcap, scn1ba, uqcc1, bahcc1a, marf1, zgc, slc16a3, cct8, ENSONIG00000021062, kitlg, nmnat1*		*mrps23, dnajc17, cab39, zgc:152 863, mpz, c2orf42, znf592, naa38*
*dmrt1*	*c7a, stxbp1a, trap1, rnf126, panx1a, smu1b, atp1b2b, rtkn, gabrb2a, LOC100690455, ENSONIG00000018490, enpp2, cdk21, itga8, ENSONIG00000031428, eri2, cyp17, srp68*		*crabp2b, polm, stox2a, mettl5, tox, znf646, smfn, mrps27, zc3h10, prdm10, rabepk, stk38l, usp20, tmem150aa, c5orf51, serpine2, dpm2, olahunc93b1*
*amh*	*cse1l, adcyap1a, sox9a, cx32.3, ovch2, rnf8, pdgfra, sash1b, angptl4, ppil1, ENSONIG00000043167, ENSONIG00000021256, rerg, meis3, slit2, wdr5, zgc:162 613, si:dkey-79d12.5*		
*sf1 (nr5a1)*	*bco2a, ENSONIG00000004889, LOC100690744, slc45a1, gas1, gpc1a, mier2, tmpoa, crip2l, st8sia5, kctd12.1, gth-rii, ENSONIG00000030718, ier2b, cadm1b, mapkapk3, rap1gap2a, LOC100695723, sfrp5*		*tent4b, kctd6a, nbr1b, psmd10, bet1, rspry1, LOC102083195, fbx046, ENSONIG00000011776, ENSONIG00000027815, parp16, trnt1, pdk3, rabep2, peli2, dusp4, cyp17a2, LOC100711445, ENSONIG00000032566*
*dax1 (nr0b1)*	*slc51a, n4bp3, cav1, ENSONIG00000032749, itk, mbnl2, sdcbp2, sorbs3, hnrnpll, ENSONIG00000012129, cbll1, scgn, ENSONIG00000035769, plat, piga, entpd1, klc1a, ENSONIG00000007130*		
*hsd11b1la*	*gtf3c5, mrpl40, mcub, tom1l2, pthlha, afap1l1a, alkal2, fgf18a, vps16, pdgfc, ENSONIG00000008822, f2rl1.2, pkdccb, hs3st1l2, olfml2a, rnf180, snai1a, stx11a*		
*hsd11b2*	*traip, enoph1, katnbl1, si:cabz01090165.1, aifm1, tcima, LOC100695259, tmem69, psmd4a, plgrkt, col14a1a, tardbpl, tmem165, itih5, fgfr1op2, eif2s2, las1(3), prep, las1(4)*		

In 15 and 40 dpf brains, where the majority of the genes were correlated with the IR, taking into account the direction of regulation—even if not significant (↑↓exp)—of these correlated genes (correlation takes priority over the direction of regulation) allowed several genes to emerge whose expression profile appears very interesting (Supplementary Fig. S3). Thus, the most interesting genes that stand out are *fam185a* (↓exp and −Corr), *lsm12b* (↓exp and −Corr), *nipal2* (↓exp and −Corr) co-expressed with the brain aromatase *cyp19a1b* (CYP19A1) in the 15 dpf brains (Supplementary Fig. S3B). In the 40 dpf brains, we found the gene *znf592* (↓exp and −Corr) co-expressed with the *cyp11c1* gene, the genes *serpine2* (↑exp and +Corr), *c5orf51* (↑exp and +Corr), *crabp2b* (↑exp and +Corr), *olah* (↑exp and +Corr), *tmem150aa* (↓exp and −Corr), *zc3h10* (↓exp and −Corr), and *znf646* (↓exp and −Corr) co-expressed with the *dmrt1* gene, the genes *kctd6a* (↑exp and +Corr), *psmd10* (↑exp and +Corr), ENSONIG00000032566 (↑exp and +Corr), ENSONIG00000027815 (↓exp and −Corr), *fbx046* (↓exp and −Corr), LOC102083195 (↓exp and −Corr), *rspry1* (↓exp and −Corr), and LOC100711445 (↓exp and −Corr) co-expressed with the *sf1* (*nr5a1*) gene (Supplementary Fig. S3C). All these genes, according to their expression profile, would be involved in either ovarian or testicular differentiation under the effect of high temperatures.

#### Kyoto Encyclopedia of Genes and Genomes Pathway in Gonads and Brains Subjected to High Temperature

Analysis of the Kyoto Encyclopedia of Genes and Genomes (KEGG) pathways common to the DEG, Corrs, and DSG genes and likely to be involved in sex determination and sex differentiation, in the gonads but also in the brains (15 dpf and 40 dpf), revealed signalling pathways related to the biosynthesis of steroid hormones, neuroactive ligand-receptor interaction, the gonadotropin hormone-releasing hormone (GnRH) signalling pathway, ovarian steroidogenesis, the oestrogen signalling pathway, thyroid hormone signalling pathway, cortisol synthesis and secretion, GnRH secretion, the mitogen-activated protein kinase (MAPK) signalling pathway, forkhead box O (FOXO) signalling pathway, mechanistic target of rapamycin (mTOR) signalling pathway, phosphatidylinositol 3-kinase (PI3K)/protein kinase B (AKT) (PI3K-AKT) signalling pathway, adenosine monophosphate (AMP)-activated protein kinase (AMPK) signalling pathway, wingless and int-1 (WNT) signalling pathway, transforming growth factor beta (TGF-BETA) signalling pathway, and focal adhesion.

In 15 dpf gonads, only 1 gene stands out in the thyroid hormone signalling pathway, MAPK signalling pathway, PI3K-AKT signalling pathway, and focal adhesion, and 2 genes stand out in the AMPK signalling pathway (Supplementary Fig. S4A) as DEG while all other genes involved in the signalling pathways at this stage were predominantly Corrs (+ and −) (Table 4). In the 40 dpf gonads, the majority of the DEGs, Corrs, and DSGs were involved in these signalling pathways, except for the one linked to GnRH secretion where very few genes were found (Table 4). At this stage, the MAPK signalling pathway involves the largest number of DEGs, with 81 DEGs (↑↓Exp) and 20 Corrs (+ or −) (Supplementary Fig. S4B), followed by the PI3K-AKT signalling pathway with 67 DEGs (↑↓Exp) and 24 Corrs (+ or −) (Table 4). In both 15 dpf and 40 dpf brains, very few genes were involved in the different signalling pathways and all were correlated (+ and −) with the IR (Table 4).

**Table 4:** KEGG pathways present in gonads and brains of fish subjected to high temperature at 15 and 40 dpf

	Gonad						Brain					
	15 dpf			40 dpf			15 dpf			40 dpf		
Descriptions	DEG	Corr	DSG	DEG	Corr	DSG	DEG	Corr	DSG	DEG	Corr	DSG
Steroid hormone biosynthesis		1(+)		30↑ 8↓	1(+) 10(−)	2		3(−)			4(−) 1(+)	
Neuroactive ligand-receptor interaction		3(+) 2(−)		41↑ 13↓	1(+) 13(−)						3(−)	
GnRH signalling pathway		4(+) 2(−)		18↑ 12↓	4(+) 5(−)	2		1(−)			3(−)	
Ovarian steroidogenesis		1(+)		19↑ 4↓	1(+) 5(−)			2(−)			4(−)	
Oestrogen signalling pathway		4(+) 3(−)		21↑ 16↓	6(+) 7(−)	1		1(+)			1(+) 3(−)	
Thyroid hormone signalling pathway	1↓	1(+) 3(−)		33↑ 18↓	4(+) 3(−)			1(+)		1↑	4(−)	
Cortisol synthesis and secretion		2(+) 1(−)		21↑ 6↓	1(+) 4(−)			1(−) 1(+)			5(−)	
GnRH secretion		1(+) 2(−)		8↑ 9↓	3(+) 5(−)						2(−)	
MAPK signalling pathway	1↑	4(+) 6(−)		46↑ 35↓	8(+) 12(−)	3		1(−)			2(+) 9(−)	
FOXO signalling pathway		6(+) 4(−)		28↑ 26↓	9(+) 9(−)						2(−)	
mTOR signalling pathway		1(+) 9(−)		27↑ 34↓	7(+) 6(−)	1		2(+) 1(−)			1(+) 1(−)	
PI3K-AKT signalling pathway	1↑	1(+) 7(−)		36↑ 31↓	8(+) 16(−)	1		3(+) 3(−)		1↑	2(+) 7(−)	
AMPK signalling pathway	2↓	1(+) 3(−)		25↑ 17↓	4(+) 5(−)	1		1(+)			6(−)	
WNT signalling pathway		4(+) 2(−)		37↑ 26↓	10(+) 4(−)	2		2(+)			3(−)	
TGF-BETA signalling pathway		6(+) 1(−)		31↑ 15↓	2(+) 11(−)	2		3(−)			6(−)	
Focal adhesion	1↑	3(+) 3(−)		32↑ 16↓	7(+) 11(−)	3		2(+) 1(−)		1↑	9(−)	

↑ Upregulated gene, ↓ downregulated gene, (+) positive correlation of gene expression with inversion rate (IR), (−) negative correlation of gene expression with inversion rate (IR), DSG: differentially spliced gene.

#### Gene Ontology Terms Associated to Genes for Which Expression Is Affected in Gonads and Brains Exposed to High Temperature

To identify the signalling pathways and processes that may play a role in sex reversal, we submitted the different gene lists (DEGs, DSGs, Corrs) to an analysis of the common GO terms. Among the three categories of GO terms available—molecular function (MF), cellular component (CC), and biological process (BP)—the BP category was revealed as the most informative. We decided to analyse the upregulated and downregulated DEGs separately, as well as the positively and negatively correlated genes. Among the DEG, Corr, and DSG, the most relevant GO terms were identified and associated with the BP category.

In 15 dpf gonads, a total of 10 (↑Exp), 11 (↓Exp), 10 (−Corr), and 13 (DSG) GO terms were identified and associated with the BP category (Fig.5A). At this stage, the most significant GO terms identified were related to cilium movement (↑Exp), cilium organization (↑Exp), pyruvate metabolic process (↓Exp), autophagy (−Corr), development of primary male sexual characteristics (DSG) (Supplementary Table S8) and male sex differentiation (DSG) (Fig.5A). In the 40 dpf gonads, a total of 17 (↑Exp), 11 (↓Exp), 1 (+Corr), and 8 (−Corr) GO terms were identified and associated with the BP category (Fig.5B). At this stage, the most significant GO terms were identified as gastrulation (↑Exp), sex determination (↑Exp and −Corr) (Supplementary Table S8), response to oestradiol (↑Exp and −Corr), cellular response to heat (↓Exp), response to heat (↓Exp) (Supplementary Table S8), and autophagy (↑Exp and +Corr) (Fig.5B).

In 15 dpf brains, a total of 10 (↓Exp) GO terms were identified and associated with the BP category (Fig.5C). At this stage, the GO terms linked to the formation of the translation pre-initiation complex (↓Exp), cellular response to cold (↓Exp), pyridine-containing compound biosynthetic process (↓Exp), and the L-serine metabolic process (↓Exp) (Fig.5C) were included as the most significant GO terms. In the 40 dpf brains, 20 (↑Exp), 3 (↓Exp), 10 (+Corr), 2 (−Corr), and 10 (DSG) GO terms were identified as associated with the BP category. Among these GO terms, the most significant were those related to visual perception (↑Exp), detection of light stimulus (↑Exp), response to light stimulus, mitochondrial translation (+Corr), cellular component assembly involved in morphogenesis (↑Exp and DSG), RNA splicing (+Corr), and the C21-steroid hormone biosynthetic process (−Corr) (Fig.5D).

## Discussion

### Effect of High Temperature on Survival and Male Rates

At the population and family levels, there is high variability in thermosensitivity that could be linked to the variable genetic potential of the parents. This variability was previously shown by Baroiller et al. (1995) [12], Baras et al. (2001) [42], Baroiller and D’Cotta (2001) [43], and Tessema et al. (2006) [16]. It is related to parental influences that would control the temperature sensitivity of *O. niloticus* from one strain to another and from one family to another. It was shown in *M. menidia* that this variability in thermosensitivity can be explained by the variation of temperature-sensitive patterns related to the geographic origin of strains [20]. The wildness of the broodstock used in the present study would influence the percentage of males obtained after thermal treatment, which could be improved in the different progenies by the effect of domestication [10, 44]. Overall, all the tested populations showed sensitivity to high temperature at different degrees. Gobé populations from the Ouémé, Togbadji, and Sohoumé populations in the Mono river basin showed the best male and sex reversal rates after high temperature treatment.

During our work on *O. niloticus*, the heat treatment was applied for 20 days to adequately cover the critical period for sex reversal, starting at 10 dpf [12–14, 16]. The results of Bezault et al. (2007) [10], Azaza et al. (2008) [45], and our results in comparison to those of Wessels and Hörstgen-Schwark (2007) [14] suggest that a treatment duration longer than 10 days covering the period of sexual differentiation likely improves the masculinization rate obtained after a thermal treatment [12, 13, 16]. In general, the survival rates recorded in our study (from 79% to 92% at the population level) for both control and treated batches are higher than those previously observed in other studies [13, 14, 45] that globally reported survival rates ranging from 60% to 81% in batches treated for 10, 21, or 28 days. In contrast to the results obtained by Baroiller et al. (1995) [12] and Wessels and Hörstgen-Schwark (2007) [14], but in line with those of Azaza et al. (2008) [45] and Pandit and Nakamura (2010) [46], the thermal treatment applied to juveniles here globally reduced the survival rate of the different treated populations. However, the existence of families with better survival rates (30% of tested families) after treatment shows that the effect on survival may vary from one progeny to another within the same population.

The percentage of males obtained in the treated batches was significantly higher than that in the control batches for all populations. A high variable sensitivity to temperature was recorded at the family and the population level. Even within the same population, we reported a high variability in thermosensitivity between families (Supplementary Fig. S1B). It is important to note that all progenies tested within the same population had no parents in common. Out of three populations showing the highest thermosensitivity (Togbadji, Gobé, and Sohoumè), the Togbadji and Sohoumè populations originated from the Mono basin while the Nangbéto population from the same basin displayed the lowest sensitivity to high temperatures with 38 ± 42% of IR. Globally, the Nangbéto population displayed particular biological characteristics with low reproductive performance [40] and lower growth performance (unpublished data). The genetic characterization of this specific population, based on Single nucleotide polymorphism technology, revealed the greatest genetic differentiation (*F_st_* from 0.091 to 0.278) compared to the other populations collected in Benin (*F_st_* from 0.018 to 0.143) [41]. These observations clearly suggest that the low temperature sensitivity displayed by this population is linked to its specific genetic differences [16], as seems to be the case for reproductive and growth performance. Unfortunately, at this stage of our work, there is no valid information to explain the low performances of the Nangbéto population (reproduction, growth, and temperature sensitivity).

It is also noteworthy that the Gobé population displayed a high percentage of males in the control group (77 ± 22%), while nevertheless also presenting among the highest inversion rates upon thermal treatment (58 ± 28%) (Table 1). Considering the families from the Gobé population, we find that the male rates in the control batches of families 1, 2, and 4 significantly deviated (69%, 100%, and 88.4%, respectively) from the expected balanced sex ratio (50:50). Thus, at the beginning of this experiment, we were already starting with a very high rate of males in the control batches. For these three Gobé families (1, 2, and 4), from the respectively 31, 0, and 34 remaining females, a huge majority of, respectively, 23, 0, and 26 were effectively reversed into neo-males which are males resulting from sexual reversal of females. Consequently, only 8, 0, and 8 of these females were able to withstand sex reversal pressure and thus maintain their original sex. We cannot say at this stage whether they were genetic XX females that resisted the masculinizing effects of high temperatures or individuals resulting from feminization of XY males [47, 48]. Also, with such a high spontaneous masculinization rate, we cannot exclude that one of the male parents in our breeding was an XX neo-male. However, such a crossing would have resulted in a purely XX offspring, theoretically all female. Possibly, Gobé family 3 was such a case with 50.4% males in the control batch. This hypothesis should not be dismissed since it was reported that XX males naturally occur in the wild [10] and the wild Gobé broodstock comes from a basin where temperatures can vary from 17.6 ± 2.5°C to 48.3 ± 2.4°C throughout the year (Agency for the Safety of Air Navigation in Africa and Madagascar, data from 2005 to 2015); we can assume that at some stage in their development cycle, these broodstock could have faced masculinizing temperatures (32°C to 36°C) [12, 45]. Thus, this broodstock would have transmitted epigenetic markers to their offspring, which would explain the high rate of spontaneous males [49] and which would have given them a high sensitivity to the effects of masculinizing temperatures. Taken together, it can therefore be concluded that the Gobé population stands out by its very high rates of spontaneous males as well as high reversal rate by temperature; compared to other populations, it has a very high susceptibility to masculinization. This susceptibility could be due to epigenetic mechanisms since it was also reported in the sole *Cynoglossus semilaevis*, for example, that pseudo-males resulting from thermal sex reversal transmitted epigenetic markers to their offspring, leading to an increase in the neo-male population by spontaneous sex reversal without temperature induction [49].

The Nangbéto family 1 and Togbadji family 2 displayed a sex reversal of 100% in favour of males, which was exceptionally high compared to our other results and to those obtained in other studies [10, 45, 50]. These results could be due to the low number of sexed fish (19 and 21, respectively), possibly as a result of the high mortality of females. However, the survival rates at 31 dpf recorded for these batches (84 and 65.3%, respectively) (Supplementary Fig. S1A) lead us to moderate this conclusion, according to which these male rates were obtained following a high mortality of the females of these families, in particular in that of Nangbéto.

### Effect of High Temperatures on the Gonad and Brain (Brain) Transcriptomes

Embryonic gonads are unique as they are the only organs that can develop into two mutually exclusive phenotypes [51]. Sex is then determined by activation of the testicular or ovarian pathway and repression of the alternative pathway, with many genes being expressed in a sexually dimorphic manner [52]. This is even more striking when genotypic sex determination (GSD) can be dominated by environmental or temperature sex determination (TSD), as seen here in Nile tilapia. In this developmental context, where many genes must be activated or repressed spatially and temporally [53], epigenetic mechanisms regulating gene expression are increasingly being studied [54].

In this study, the effects of high temperature on sex determination and differentiation processes were exploited to investigate the effect of the environment on gonadal and cerebral (brain) gene expression patterns in fish. We showed that the phenotypic results obtained after high temperature treatment are the result of an observed shift in the expression of a number of genes and signalling pathways previously shown to be involved in sex determination and differentiation in the Nile tilapia. At the family level, this masculinization was not complete, as we still observe resistant females in most treated batches, which may cause a weaker transcriptional response. However, we can assume that the observed changes in gene expression will tip the balance towards a male outcome.

We observed that the progenies subjected to high temperature displayed a different gonadal transcriptome at 15 dpf compared to 40 dpf, which had many more DEGs, Corrs, and DSGs (Fig. 2). In the gonads, samples at 40 dpf revealed a significant increase in the number of DEGs, Corrs, and DSGs with, respectively, 12 955, 1120, and 189 more genes compared to 15 dpf samples. Focusing on genes that were previously identified as being involved in sexual differentiation in fish (Supplementary Table S5) [31, 55–59], it appears that significant upregulation of genes involved in testis differentiation, such as *dmrt1, amh, cyp11c1*, and *dax1*, was observed at 40 dpf (Fig.2E) while genes involved in female differentiation (*cyp19a1a* and *foxl2*) were detected but were not significantly regulated (Fig. 2E). In 15 dpf gonads, although mRNA for these genes was detected, their expression did not vary significantly in treated batches compared to control batches (Fig. 2B). These results indicate that, although the process of sex reversal is probably already initiated at 15 dpf [60], sexual differentiation has not yet taken place at this stage. Also worth noting is the detection of brain aromatase (*cyp19a1b*) expression in 15 dpf gonads and its upregulation in the 40 dpf gonads after high temperature treatment, while that of the gonadal aromatase (*cyp19a1a*) remained unchanged. Expression of *cyp19a1b* in gonads was previously reported by Jørgensen et al. (2008) [60], who detected the expression of brain aromatase (*cyp19a1b*) in the gonads of zebrafish. A question then arises regarding the role of this enzyme in the gonads, especially in a context of masculinization.

**Figure 6:**
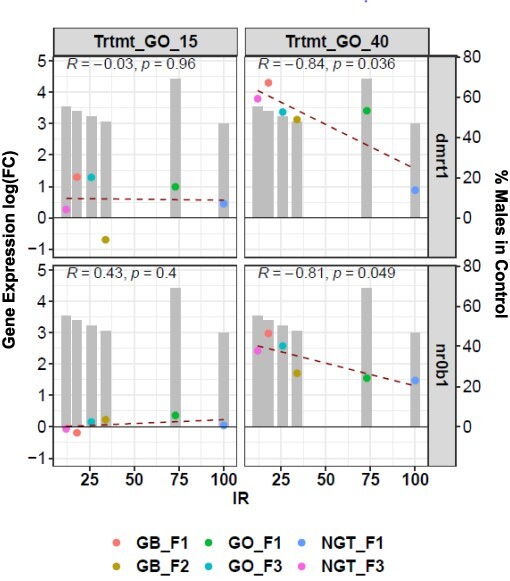
Illustration of the correlation of *dmrt1* and *dax1* (*nr0b1*) gene expression with IR as a function of male rates in control batches. At 40 dpf in the gonads, families with high rates of males in the control batches had low levels of overexpression of the *dmrt1* and *dax1* genes

Interestingly, in the 40 dpf gonads, the *dmrt1* and *dax1* genes involved in the male pathways were clearly upregulated in treated animals, among others; however, their fold-induction rates were negatively correlated with the rate of sex reversal (IR). Thus, the higher the reversal rate to males, the lower the level of overexpression of the *dmrt1* and *dax1* genes at 40 dpf. Such a counterintuitive result may actually be linked to the observed correlation between spontaneous sex inversion and temperature-dependent sex inversion. Populations with a high predisposition for spontaneous sex inversion have a higher expression of the male differentiation genes *dmrt1* and *dax1* in controls; thus, a weaker induction is required to cause further sex inversion upon temperature treatment (Fig. 6).

In brains, none of the genes known for their involvement in sexual differentiation was significantly differentially expressed. However, expression of the brain aromatase gene *cyp19a1b* was negatively correlated with IR in 15 dpf brains. Therefore, as the sex reversal rate increased, the level of upregulated expression of this gene was low. Similarly, in brains at 40 dpf, *foxl2* mRNA was detected with a negative correlation with IR. This gene was reported to be expressed in the brain of tilapia in adulthood [61], but results from Gennotte et al. (2014) [62] (14 dpf) and our results (40 dpf) show that the *foxl2* gene is expressed earlier in fingerling brains (certainly in the brain). This reinforces the hypothesis of early brain sexualization that has already been suggested by Gennotte et al. (2014) [62] and Rougeot et al. (2008) [63] in *O. niloticus*, and by Santi et al. [22] in the African catfish *Clarias gariepinus*. Interestingly, in the brains at 40 dpf, the *dmrt1* gene was detected with expression that was positively correlated with IR. *dmrt1* expression in the brains of African catfish (*Clarias gariepinus*) was previously reported [22], further supporting the notion that this gene is also expressed in the brain rather than exclusively in gonads.

When we filtered our RNA-seq data for DEGs and Corrs, in 15 dpf gonads, we identified the *kmt5c* (lysine methyltransferase 5C), *abhd4* (abhydrolase domain containing 4), and *osbpl3b* (oxysterol binding protein like 3b) genes (Supplementary Table S6). *kmt5c* (↑Exp and +Corr) is believed to be strongly expressed in the testes compared to the ovaries, and specifically methylates, the monomethylated and dimethylated lysine 20 (Lys-20) (H4K20me1, H4K20me2) of histone H4, to produce, respectively, dimethylated Lys-20 (H4K20me2) and trimethylated Lys-20 (H4K20me3) [64, 65]. Trimethylation of H4 Lys-20 represents a specific marker for epigenetic repression of transcription [64]. DNA methylation is often proposed to be involved in sex reversal in fish [66–68]. Thus, through histone methylation and its associated DNA methylation, the *kmt5c* gene could be involved in the regulation of gene expression during temperature treatment, leading to masculinization at later stages. The *abhd4* gene (↓Exp and −Corr), in contrast, is reported to be highly expressed in ovaries relative to testes. Although largely understudied, *abhd4* has recently been suggested to play a role in tumour suppression by limiting proliferation and the cell cycle [69], while *abhd4* knockdown confers resistance to anoikis (a specific form of apoptosis) in RWPE1 immortalized prostate cells and *abhd4* over-expression of *abhd4* increases sensitivity to anoikis [70]. Thus, one could suggest that *abhd4* downregulation would play a role in gonadal differentiation by conferring resistance to apoptosis of primordial germ cells (PGCs) for ovarian differentiation. During the process of gonadal differentiation, an early distinction can generally be made between male and female gonads by the number of PGCs. Ovarian differentiation is generally associated with a higher number and greater proliferation of PGCs than testicular differentiation. In some heat-sensitive species, high masculinizing temperatures induce a reduction in the number of PGCs by apoptosis [71–73]. Thus, the observed downregulation of *abhd4* would induce resistance of PGCs to apoptosis, therefore leading to resistance to testicular differentiation in some individuals in the treated batches and would explain the existence of females resistant to the action of high temperatures during the sexual differentiation period. Another hypothesis is that the action of *abhd4* could be involved in the feminizing effect of temperature reported in previous studies [47, 48].

In 15 dpf brains, we also identified the *aebp2* (↓Exp and +Corr) and *jarid2* (↑Exp and No_Corr) genes whose expression is simultaneously modified in brains. The *Aebp2* and *Jarid2* proteins define a distinct class of proteins that present colocalization with polycomb repressor complex 2 (PRC2) on chromatin and play a pleiotropic role in embryonic development [74]. They recruit the PRC2 complex, which is thought to repress transcription by methylating lysine 27 on histone H3 [75]. Due to this recruitment capacity, *JARID2* is involved in transcriptional regulation and differentiation of embryonic stem cells (ES) [76]. A previous study reported that, in response to temperature, the expression of both genes could influence the expression of many other genes via epigenetic modifications. Thus, taken together, *aebp2* and *jarid2* could potentially interact as new sex-determining genes in TSD vertebrates such as the snapping turtle (*Chelydra serpentina*) [77]. Further studies will be required to understand the roles of the *kmt5c, abhd4* (in 15 dpf gonads), *aebp2*, and *jarid2* (in 15 dpf brains) genes in sex determination and differentiation in tilapia.

Interestingly, we have found co-expressed genes of interest associated with the *cyp19a1b* gene in 15 dpf brains and with the *dmrt1, cyp11c1*, and *sf1* genes in 40 dpf brains. Analysis of these co-expression networks and the expression profiles of some genes in the brains suggests that the gene cluster *fam185a*/*lsm12b*/*nipal2,* all (↓exp and −Corr) and co-expressed with *cyp19a1b* (CYP19A1) at 15 dpf (Supplementary Fig. S3B), may be involved in female differentiation. In the 40 dpf brains, the gene clusters *tmem150aa*/*zc3h10*/*znf646* (all ↓exp and −Corr), *fbx046*/LOC102083195/*rspry1*/LOC100711445/ENSONIG00000027815 (all ↓exp and −Corr), and the *znf592* gene (↓exp and −Corr), respectively, co-expressed with *dmrt1, sf1*, and *cyp11c1*, may also be involved in female differentiation (Supplementary Fig. S3C). In contrast, still in 40 dpf brains, the gene clusters *serpin2*/*c5orf51*/*crabp2b*/*olah* (all ↑exp and +Corr), *kctd6a*/*psmd10*/ENSONIG00000032566 (all ↑exp and +Corr), respectively, co-expressed with the *dmrt1* and *sf1* genes, are involved in male differentiation. Previous studies have shown, for example, that the *serpin2* (serpin family E member 2) gene encodes a group of proteins that inhibit serine proteases and may also influence the establishment of the testis pathway in vertebrates [52, 78]. *crabp2b* (cellular retinoic acid binding protein 2b) is involved in the morphogenesis of the hindbrain and the regulation of the retinoic acid receptor signalling pathway during embryonic development and it is thought to stimulate spermatogonial development in zebrafish through an increase in germ cell proliferation activity [79]. The *psmd10* (26S proteasome non-ATPase regulatory subunit 10) gene is supposed to be involved in cell proliferation and testicular differentiation and was proposed as a biomarker to detect the disruption of testis differentiation by oestrogenic endocrine disruptors in *Xenopus laevis* [80].

The most relevant GO terms DEG, DSG, and Corr were identified and associated with the BP category, suggesting that the biosynthesis of certain compounds associated with the various GO terms was impacted. In the 15 dpf gonads, we observed higher expression of genes dependent on the GO terms related to cilium (movement, assembly, and organization) [81, 82] and lower expression of genes dependent on the GO terms associated with purine biosynthesis. Cilium formation was identified as a key signalling coordinator during organogenesis and remains present in different gonadal cell types. It may be important for gonad cell differentiation and gonadal sex differentiation in mammals [83, 84]. However, the role of primary cilium formation in sex determination and gonadal differentiation in fish is completely unknown. Purines are known to play a key role in neurotransmission and neuromodulation [85]. In 40 dpf gonads, GO terms such as sex determination, autophagy, and gastrulation were upregulated [86]. Upregulated genes identified in the 40 dpf gonads are candidates for a molecular network of sex differentiation. Thus, the main genes associated with these GO terms and involved in sex differentiation at 40 dpf have their expression profile maintained at this stage. This result is confirmed by the co-expression networks of genes associated with *amh, dmrt1, cyp11c1, hsd11b1la*, and *hsd11b2* genes observed at 40 dpf. In contrast, we observe at this stage a downregulation of GO terms associated with the response to heat. Thus, in 40 dpf gonads, we would see a return to normal of the stress response process induced by the high temperature treatment. In 15 dpf brains, we observed the GO terms associated with male sex determination whose gene expression was positively correlated to IR and the GO terms associated with the steroid hormone biosynthetic process whose gene expression was negatively correlated with IR. These results suggest that sex determination takes place in the brains (certainly in the brain) between the start of treatment (10 dpf) and 15 dpf, while sex differentiation started between 15 dpf and 30 dpf (end of treatment) and was maintained up to 40 dpf.

The DEGs, Corrs, and DSGs were most enriched in MAPK, PI3K-AKT, WNT, steroid hormone biosynthesis, neuroactive ligand-receptor interaction, GnRH, ovarian steroidogenesis, oestrogen, thyroid hormone and cortisol synthesis, and secretion signalling pathways in the 40 dpf gonads. Previous studies have shown that activation of MAP kinase pathways is required for cell proliferation and also activates appropriate downstream targets involved in physiological acclimatization [87, 88]. Also, WNT signalling pathways play a role in carcinogenesis and embryonic development [89], while mTOR signalling pathways play important roles in response to stress, including activation of autophagy [90] and modulation of protein synthesis [91]. These responses conserve energy and promote survival during prolonged periods of stress. Pathways involving steroid hormones are believed to regulate various physiological functions such as reproduction, blood salt balance, maintenance of secondary sexual characteristics, stress response, neuronal function, and various metabolic processes [92]. In teleosts, the neuroactive ligand-receptor pathway was reported to play an important role in the reproduction and gonadal development of Nile tilapia and yellow perch [93, 94]. It has been suggested that this pathway may serve as a key modulator in the nervous and reproductive systems to control the production of sex hormones [81]. Cortisol is a hormone associated with stress and regulates many physiological processes. Cortisol levels are elevated in fish exposed to high temperatures or high stocking density in tilapia, zebrafish, and brook trout (*Salvelinus fontinalis*) [95–98]. In addition, cortisol is linked to masculinization in some fish species such as pejerrey, medaka, and Japanese flounder [99–101]. Enrichment of pathways involved in steroid hormone and cortisol synthesis and secretion suggests that cortisol may interact with steroidogenesis to facilitate high-temperature sex reversal [102–104]. Thus DEGS, DGS, and Corrs enriched in all these signalling pathways are involved in sex determination and differentiation, gonadal development, growth, and acclimatization to high temperature-induced environmental change.

From our results, it appears that high temperatures influenced the male rates of the different *O. niloticus* populations. This effect was variable from one population to another and within the different populations from one family to another. Transcriptome analysis of gonad and brain samples collected during the reversal phase allowed an investigation of the genes involved in biological cascades inducing sex determinism in Nile tilapia. GO terms associated with autophagy, sex determination, male sex determination, male gonad development, response to oestradiol, and male sex differentiation were observed, confirming the implementation of the different processes necessary for sex reversal noted through the first part of our work. Then, a series of BPs were activated: steroid hormone biosynthesis, neuroactive ligand-receptor interaction, GnRH signalling pathway, ovarian steroidogenesis, MAPK signalling pathway, PI3K-AKT signalling pathway, WNT signalling pathway, oestrogen signalling pathway, thyroid hormone signalling pathway, cortisol synthesis and secretion, and GnRH secretion. This leads to changes in the relative amount of sex steroid hormones (androgens and oestrogens), which promotes the development of neo-males in *O. niloticus*. Our RNA-seq data also revealed a complex of genes (*kmt5c, aebp2, abhd4*, and *jarid2*) that may warrant further study in order to provide information on their roles in sex differentiation and in the resistance of some females to the effects of high temperatures through the phenomenon of apoptosis.

## Materials and Methods

### Origin and Broodstock Selection

*Oreochromis niloticus* broodstocks were collected in Sohoumè (SH) [X: 370 050, Y: 717 554], Nangbéto (NGT) [X: 326 763, Y: 823 660], Togbadji (TG) [X: 356 686, Y: 744 835] in the Mono river basin, Gobé (GO) [X: 430 018, Y: 886 561] in the Ouémé river basin, and Gbassa (GB) [X: 412 808, Y: 1 229 684] in the Niger river basin as described by Fagbémi et al. (2021) [41]. The different collected populations were acclimatized for 10 months from February to November 2017 in a recirculating system in the Laboratory of Hydrobiology and Aquaculture of the University of Abomey-Calavi (Benin Republic).

Ten wild phenotypic females with an average weight of 325 g ± 10.2 and five wild phenotypic males with an average weight of 412 g ± 8.7 were selected from each population. Broodstocks were housed separately according to sex and origin in 1.9 m^3^ fiberglass breeding tanks to avoid anarchic mating. Batches were fed daily at 5% of the total biomass per tank with commercial feed (BioMar, 35% protein and 6% lipid) for one week before the start of the experiment.

### Breeding, Incubation of Eggs, and Setting up Tests

After one week of feeding, one male was selected from the males’ tank and introduced into the females’ tank for natural reproduction. After introduction of the male, continuous monitoring was carried out in order to observe mating and identify the mouth-incubating female. Just after reproduction, the male was pit-tagged and isolated. The female was kept in the tank for egg mouth-incubation until 5 dpf, when the eggs were collected, the female pit-tagged and isolated. The eggs were transferred in Zug bottles for incubation. At 9 dpf, larvae from each spawn were divided into two batches, distributed into two 50 L aquariums, and maintained at room temperature (30°C). At 10 dpf, the water temperature in one of the aquariums was progressively increased by 2°C/hour (to avoid thermal shock) by heating resistors and thermostats (Biotherm 2000) to the masculinization temperature of 36.5°C for the treated batch [12] and maintained at room temperature (30°C) for the control batch until 30 dpf. Before and after treatment, the rearing temperature was maintained at 30°C. Within a spawn, the number of larvae was the same for both batches, but varied between families. Three to five families were tested per population.

### Larval Rearing and Sex Ratio Assessment

Ten dpf larvae were reared at a density ranging from 3 to 10 larvae/L depending on the hatching rates. During the treatment process, aquariums were continuously supplied with water at a temperature between 30°C and 32°C. Larvae were fed *ad libitum* four times a day. At 31 dpf, all fry were counted in order to calculate the survival rate at the end of the temperature treatment. Fry were then distributed into 50 L aquariums at a reduced density in order to maintain good rearing conditions. Fry were reared up to 90 dpf until sex ratio assessment [105]. Then, 19 to 319 individuals per batch were euthanized with an overdose (200 mg/L) of benzocaine (Sigma-Aldrich) and sex ratio determined according to the gonadic squash method described by Guerrero and Shelton [106].

### Sampling at 15 Dpf and 40 Dpf

From each batch, 25 to 100 individuals at 15 dpf and 15 to 50 individuals at 40 dpf were sampled according to the available number in the aquarium, euthanized with an overdose of benzocaine (Sigma-Aldrich), decapitated, pooled, and then preserved in RNA-preserving solution prepared in the laboratory (https://www.lifescience.net/protocols/927/rnalater/). The targeted tissues for these different samplings were gonads and brains through the heads. All samples were first stored for 24 hours at 4°C and then at −20°C during the conservation phase. Samples were transferred to the GIGA Organogenesis and Regeneration Laboratory (University of Liège) in Belgium for transcriptomic analyses.

### Transcriptomic Analysis of Gonads and Brains

#### RNA Extraction, cDNA Library Construction, and Illumina Sequencing

All tested progenies were sampled, but only six families (GB_F1, GB_F2, GO_F1, GO_F3, NGT_F1, and NGT_F3) with high and average sex reversal rates (43.8% ± 34.9) were selected for transcriptomic studies for a total of 48 samples (control and treated per family at 15 dpf and 40 dpf for gonads and brains) (Supplementary Table S1). Since the objective is not a comparative study of the transcriptome of each population, each sample from a population will be considered as a sample, so *n* = 6.

Trunks were dissected under a magnifying glass, and the gonads were removed and pooled according to the experimental batch in liquid nitrogen and then stored at −80°C. Similarly, brains of the different samples were grouped by batch, ground in liquid nitrogen and then stored at −80°C. Based on the extraction protocol of the All Prep DNA/RNA Kits (QIAGEN®, Venlo, Netherlands), Micro for gonads and Mini for the brains, total RNA was extracted from the pooled gonads and pooled brains for each batch. RNA from the gonad and brain samples was eluted in 22 and 20 μl of nuclease-free water, respectively. A DNase-I treatment was also performed during the extraction according to the recommendations of the kit. After extraction, purification by lithium chloride precipitation was performed by adding 33.3 μl of LiCL, centrifugation at 4°C for 30 minutes, and dilution of the RNA pellet in 25 μl of nuclease-free water. The quantity and quality of the RNA samples were determined using a Nanodrop ND-1000 (ISOGEN Life Science, Netherlands) and an Agilent 2100 Bioanalyzer. Samples with RNA integrity number ≥6 were used to prepare RNA libraries.

Equal concentrations of RNA were used for mRNA isolation, cDNA synthesis, and sequencing. The libraries were constructed using the Illumina® TrueSeq Stranded mRNA library method according to the TrueSeq RNA Sample Preparation guide (Illumina Technologies, San Diego, CA, USA). Once the RNA libraries were made, the cDNAs obtained by mRNA reverse transcription were amplified using 15 PCR cycles before being sequenced. An analysis of the quality of the libraries was performed using a Nanodrop ND-1000 (ISOGEN Life Science, Netherlands), followed by an electropherogram for each library. These different analyses allowed us to see the distribution of fragment sizes (Qiaxcel, Qiagen). A qPCR was also realized to determine the exact concentration of each library. These quality control (QC) analyses were performed at the GIGA-Research Institute’s Genomics Technology Platform. Paired-end sequencing was done using NovaSeq (Illumina Novaseq 6000) to obtain reads of approximately 150 base pairs (bp) in length. In total, 48 cDNA libraries were constructed from the 48 samples.

#### Alignment and General QC

The data obtained at this stage were the reconstructed reads and their unique quality code for each position, delivered by the sequencer. These data were provided in fastq.gz compressed format and were further processed according to the flow chart presented in Supplementary Fig. S5

The raw sequence reads were aligned on the Nile tilapia genome (O. niloticus UMD NMBU, Maryland University, June 2018) following the method described by Conte et al. (2017) [107] and using the STAR v.2.7.3a program [108]. This analysis generates an alignment file in Bam format, featuring in a matrix for each gene the read count and various alignment QC files (Supplementary Fig. S2). From this alignment file, a sequencing QC report using FASTQC v.0.11.9 [109] was generated and consolidated using multiQC software into a single report [110] to evaluate the general qualitative and quantitative sequencing and alignment parameters for each sample. The quality of the extracted RNA was further evaluated by generating two additional quality reports, namely the Gene Body Coverage and the TIN score (transcript integrity number). Both QC analyses were performed using RSEQC v.2.3.2 software and the Consortium of high-performance computing (CECI) cluster [111].

#### Differentially Expressed Genes

Differential expression data were obtained using Limma-voom software based on data from the gene count matrix [112–114]. Analysis of the relative expression difference between high temperature treated (Ltherm) and control batches was performed for all stages, tissues, and families. Control and Ltherm batches were matched for this analysis by experimental design. The data from this analysis are presented in tabular form, where the relative differential expression is expressed in logarithmic base two with an associated *P*-value. Only genes for which the difference in expression, whether by induction or repression, is significant (*P*-value ≤ 0.05) were considered to be DEGs.

#### Differentially Spliced Genes

Similar to DEGs, we evaluated the difference in expression at the level of each exon for each gene in order to identify different isoforms that would be a function of the treatment, ultimately reflecting potential alternative splicing. This analysis required the generation of a counting matrix where the unit is not the gene but the exon. This was performed on the CECI computing cluster using Subread v.2.0.0 software [115]. Differential expression analysis of the exons at the level of each gene was then performed using Limma-voom software [112–114]. This analysis was performed on the basis of comparisons between treated (Ltherm) and control batches, considered to be matched within each family, as for the DEGs. The data resulting from this analysis show the genes for which there is a significant difference in the expression of at least one exon between conditions (FDR ≤ 0.05).

#### Genes Whose Expression Is Correlated to Reversal Rate

Based on the read count for each gene and sample, we analysed the genes for which expression was correlated with the reversal rate observed in each clutch. For each family, we generated the individual relative expression rate between treated batches and their respective controls. The first step was normalization of the count tables according to library sizes using the CPM function of the edgeR package [116]. Then, for each family, the log2 (Ltherm/Control) count ratio was calculated for each gene. From this matrix, we focused on the genes with a correlation between this value and the family’s reversal rate. This step was performed using the WGCNA package [117].

#### Co-expressed Gene Networks

The same Ltherm/Control ratio matrix was used to classify genes that are co-regulated in the different families as a consequence of thermal treatment. Using the WGCNA package [117], we obtained a similar matrix (adjacency matrix) in which each cell represents a numerical value for each gene combination representing the degree of co-expression. Selected genes were graphically represented as a network using the iGraph and ggraph packages in R software [118, 119].

#### Pathway Enrichment Analysis

The transcriptomic data analysis essentially resulted in lists of genes of particular interest, DEGs, DSGs, Corr, or Co-expressed Gene Networks (Co-exp). Pathway enrichment analysis uses gene ontology (GO) databases, such as Kyoto Encyclopedia of Genes and Genomes (KEGG) or GO terms, to identify specific gene networks or regulatory pathways of the genes belonging to the list. This allows us to identify regulatory pathways or functions that are specifically impacted by the investigated treatment.

Enrichment analysis of the pathways present in the KEGG database [120, 121] was performed using the KEGG enrichment function of the clusterProfiler package [122]. We used the Orthology database (KEGG Orthology, KO), requiring the transformation of all gene identifiers from the Ensembl database into NCBI identifiers [123]. In that process, we strongly limited orthology redundancies by selecting only the identifier associations for which the association was most frequently referenced among the NCBI and Ensembl gtf annotation files and the BiomartR database [124]. The translated NCBI identifiers could be associated at the level of the KEGG database with an orthology identifier (KO). The different lists of KOs (DEG, DSG, or Corr) were subjected to an enrichment test and the enrichment was visualized as a whole using a dot plot [122]. Some pre-selected pathways of interest were also used to graphically visualize the genes of interest and their interactions. In addition, for each gene, the observed features (DEG, DSG, or Corr) were indicated by colour coding, splicing the rectangle into 3 distinct sections representing, in order: DEG|Corr|DSG using the pathview package [125].

#### Enrichment Analysis in GO Terms

Enrichment analysis was performed using the GO database [126–128]. The GO annotation is initially provided for each tilapia gene in the Ensembl database; however, after evaluation, some well-known genes did not have associated GO annotations. We, therefore, expanded our GO database based on genes for which human orthologues exist and which are much better annotated. Once our own GO database was generated, we analysed the enrichment of GO terms belonging to the category of BPs using the enrichGO function of the ClusterProfiler package [122]. We then categorized our gene lists according to whether the DEGs were upregulated (↑Exp) or downregulated (↓Exp) during or after high temperature treatment, or whether their expression correlated positively (+Corr) or negatively (−Corr) with IR. The results of these enrichment analyses in GO terms are also presented as a dot plot.

### Statistical Analyses

#### Sex Ratio and Survival Rate Data

For each family, the sex ratio was expressed as the proportion (%) of males relative to the total number of sexed fish. The effect of temperature on the sex ratio was expressed through the determination of reversal rate (IR) specific to each family and each population, and expressed as


$${\bf{IR}}{\mathrm{ }}\left( \% \right) = [({\bf{S}}{{\bf{R}}_{{\bf{36}}^\circ {\bf{C}}}}\unicode{x2642} - {\bf{S}}{{\bf{R}}_{{\bf{30}}^\circ }}\unicode{x2642})/({\bf{1}}-{\bf{S}}{{\bf{R}}_{{\bf{30}}^\circ }}\unicode{x2642})] \times {\bf{100}}$$

where SR_36°C_♂ is the percentage of males in the treated batch and SR_30°_♂ the percentage of males in the control batch.

At the end of the treatment period (31 dpf), the survival rate of each batch was calculated. The RSR was determined as


$${\bf{RSR}}{\mathrm{ }}\left( \% \right) = [({\bf{Srvl}}{{\bf{R}}_{{\bf{36}}^\circ {\bf{C}}}}\unicode{x2642} - {\bf{Srvl}}{{\bf{R}}_{{\bf{30}}^\circ }}\unicode{x2642})/({\bf{1}}-{\bf{Srvl}}{{\bf{R}}_{{\bf{30}}^\circ }}\unicode{x2642})] \times {\bf{100}}$$

where SrvlR_36°C_♂ is the survival rate in the treated batch and SrvlR_30°_♂ the survival rate in the control batch.

Statistical analysis was performed at three levels: within each family, within each population encompassing all families issuing from the same location, and finally at the all-populations level. An intra-family comparison of male ratio and survival rates in control versus treated batches was performed using the exact Fisher test, while the binomial test compared the sex ratio obtained in the control batches to the expected theoretical sex ratio (50:50). At the population level, the male rates and the survival rates were compared between the different batches by generating a generalized linear model (logistic regression). The correlation between RSRs and the reversal rates of different populations was tested by Spearman’s non-parametric correlation test. Statistical analyses were performed with R v3.4.4. The statistical difference threshold was defined for the value *P* ≤ 0.05. All figures were generated using the ggplot2 R package [129].

## Supplementary Material

dvad009_Supp
