# Supplementary material for: Effect of high temperatures on sex ratio and differential expression analysis (RNA-seq) of sex-determining genes in Oreochromis niloticus from different river basins in Benin
Source: Environ Epigenet. 2024 Jan 13;9(1):dvad009. doi: 10.1093/eep/dvad009 (PMC10939319; doi:10.1093/eep/dvad009)
Supplement: dvad009_Supp [file dvad009_supp.zip › suppl_data/List of Figures, Tables, and Supplementary Files.docx]

**Figures, Tables, and Supplementary Files:**

**Fig. 1:** Survival rates (A) and sex ratios (B) in control (blue) and high temperature treated (red) batches for each population. Median values are indicated by a vertical line, the boxes encompass the 25 and 75% quartiles, the dots represent the values for each individual batch, while the lines joining two dots link the corresponding control and treated batches of the same family. GB: Gbassa, NGT: Nangbéto, SH: Sohoumè, TG: Togbadji.

Fig. 2: Comparison of gene expression in gonads of high temperature-treated juveniles to that of controls (Ltherm versus control) at 15 dpf (A-C) and 40 dpf (D-F). For each stage, the data are presented as a Venn diagram, a volcano plot, and a corr-plot. (A, D) Venn diagrams representing the number of Differentially expressed genes (yellow), Differentially spliced genes (purple), and Corrs (green), respectively, as well as the number of genes common to the different categories. (B, E) Volcano plot representing the log2-fold change in gene expression versus statistical significance (−log10 of adjusted p-value). Coloured dots represent the significantly regulated genes (p-value < 0.05), red for upregulated, blue for downregulated. (C, F) Plot representing the log2-fold induction versus the correlation coefficient of this log2-fold change with the reversal rate (IR) displayed by the different families. Significant correlation is shown by the coloured dots, positively correlated in red, negatively in blue. Rectangles contain the names of genes that were selected from the literature as being relevant to sexual differentiation and maturation, the colour of the rectangle indicates significance, either for differential expression or correlation with IR.

Fig. 3: Comparison of gene expression in heads of high temperature-treated juveniles to that of controls (Ltherm versus control) at 15 dpf (A-B) and 40 dpf (C-D). For each stage, the data are presented as a Venn diagram (A, C) and a Corr-plots. (B, D). Venn diagrams representing the number of Differentially expressed genes (yellow), Differentially spliced genes (purple), and Corrs (green), respectively, as well as the number of genes common to the different categories. (B, D) Plot representing the log2-fold induction versus the correlation coefficient of this log2-fold change with the reversal rate (IR) displayed by the different families. Significant correlation is shown by the coloured dots, positively correlated in red, negatively in blue. Rectangles contain the names of genes that were selected from the literature as being relevant to sexual differentiation and maturation, the colour of the rectangle indicates significance for correlation with IR.

**Fig. 4:** Networks of genes co-expressed with the *amh*, *cyp11c1*, *cyp19a1a*, *dmrt1*, *hsd11b1la*, and *hsd11b2* genes in the 40 dpf gonads (A), with the *cyp19a1b* gene in the 15 dpf brains (B) and with the *cyp11c1* and *dmrt1* genes in the 40 dpf brains (C). Genes with red centres denote upregulated expression and blue centres denote downregulated expression. The colour of the edges of the gene indicates a correlation of the gene expression with inversion rate (IR), red for a positive correlation and blue for a negative correlation. When a gene has no colouration then it is neither a differentially expressed gene (DEG) nor is its expression correlated to the IR (Corr) in our results but belongs to the network of genes co-expressed with our gene of interest. The thickness of the lines denotes the intensity of the links between the different genes of the network.

**Fig. 5:** List of the most significant Gene Ontology (GO) terms and function of DEGs, Corrs, and DSGs associated with the effect of high temperatures in 15 dpf (A) and 40 dpf (B) gonads and in 15 dpf (C) and 40 dpf brains (D).

**Fig. 6:** Illustration of the correlation of *dmrt1* and *dax1* (*nr0b1*) gene expression with IR as a function of male rates in control batches. At 40 dpf in the gonads, families with high rates of males in the control batches had low levels of overexpression of the *dmrt1* and *dax1* genes

**Table 1**: Means and median values of male rates in each population.

**Table 2:** Correlation between relative survival rate (RSR) and reversal rate (IR)

**Table 3:** List of genes co-expressed in co-expression networks with genes of interest identified in gonads and brains at 15 and 40 dpf

**Table 4:** Kyoto Encyclopedia of Genes and Genomes (KEGG) pathways present in gonads and brains of fish subjected to high temperature at 15 and 40 dpf

**Supplementary_Table 1:** Survival (%Alive), male (%Male) and inversion rates (IR) data for control and treated batches at the family level. N_Total: Initial Number, N_Alive: Number of Survivors, N_Sexed: Number of Sexed Individuals, N_Male: Number of Males.

Supplementary_Table 2: Mean survival rates and median values on control and high temperature treated batches. **N family:** Number of family tested, **N:** Number of individuals, **Group:** Results of statistical tests of significance between control and treated batches.

**Supplementary_Table** **3**: Correlation between the male rates (Male %) and survival (Alive % 31 dpf) in control (Ctrl) and treated (Lther) batches of different populations

**Supplementary_Table** **4**: Characteristics of RNA-seq data

**Supplementary_Table 5**: List of some genes of interest from the literature

**Supplementary_Table 6:** List of eight genes identified as DEG, Corr in gonads (15 dpf) and brains respectively at 15 dpf and 40 dpf and list of eleven genes identified as DEG, No_Corr in brains (15 dpf)

**Supplementary_Table 7:** List of 75 DEGs and Corrs genes in the gonads at 40 Dpf

**Supplementary_Table 8:** Lists of genes involved in three GO terms in the gonads at 15 and 40 dpf

**Supplementary_Fig 1:** Distribution of survival rates (A) and male ratio (B) recorded by family (in column) for each population (in line). For (A) the blue asterisk on a graph shows a significant difference between the survival rates of the control and the treated batches (Ltherm). For (B) the blue asterisk on a graph shows a significant difference between the male ratio of the control and the treated (Ltherm) batches, while the red asterisk marks a significant deviation of the ratio of males from the control batch compared to the expected theoretical male ratio (50:50). GB: Gbassa, NGT: Nangbéto, SH: Sohoumè, TG: Togbadji. (*P ˂ 0.05; **P ˂ 0.01; ***P ˂ 0.001)

**Supplementary_Fig 2**: (A) Transcript Integrity Number (TIN), RNA Integrity Number (RIN) score of RNA-seq data. The TIN score is an associated numerical value ranging from 0 (complete degradation) to 100 (homogeneous distribution of reads) for each annotated gene in the reference genome, while the RIN score assigns a value from 1 to 10 to an electropherogram, 10 being the least degraded value; and (B) Gene Body Coverage. It graphically represents the coverage density of the reads aligned on average over the length of the genes in the 5'-P to 3'-OH direction. If RNA degradation is present, asymmetry appears with an overrepresentation of the 3'-OH ends.

**Supplementary_Fig 3:** Networks of most interesting co-expressed genes in gonads at 40 dpf and brains at 15 and 40 dpf. Genes with a red center staining for up expression and blue center staining for down expression. A staining of the edges of the gene marks a correlation of the gene expression with IR in red for a positive correlation and in blue for a negative correlation. When a gene has no staining then it is neither DEG nor Corr in our results but belongs to the network of genes co-expressed with our interest gene. Genes with red asterisks are those exclusively Corr (+ or -) and whose non-significant regulation direction was taken into account. The thickness of the lines of the network marks the intensity of the links between the different genes of the network.

**Supplementary_Fig 4:** KEGG pathways common to the DEG, Corrs and DSG genes in gonads at 15 dpf (A) and 40 dpf (B). The measured parameters were added by cutting the rectangles representing the genes into 3 distinct sections representing in order: DEG|Corr|DSG. The sections colored in gray show no change in the parameter according to the conditions being compared and when the gene rectangle is not colored (white), it means that no orthology at the KEGG database level has been identified among the tilapia genes.

**Supplementary_Fig. 5:** Flow chart of bioinformatics analysis. From top to bottom, starting with the raw sequencing data (blue) that were analysed using the indicated software (light red) to generate quality control (QC) and genome mapping result files (white). These intermediary files were then used in further analysis to generate the final data files (orange). Each light red box contains the information of the process performed and the software used, while white and orange boxes indicate the file format and the information given in the result files. Some final result files, such as differentially expressed genes (DEGs), can be used for further analysis, such as correlated or co-expressed genes or pathway analysis (see below).
