# Supplementary figures and images for: Effect of high temperatures on sex ratio and differential expression analysis (RNA-seq) of sex-determining genes in Oreochromis niloticus from different river basins in Benin
Source: Environ Epigenet. 2024 Jan 13;9(1):dvad009. doi: 10.1093/eep/dvad009 (PMC10939319; doi:10.1093/eep/dvad009)

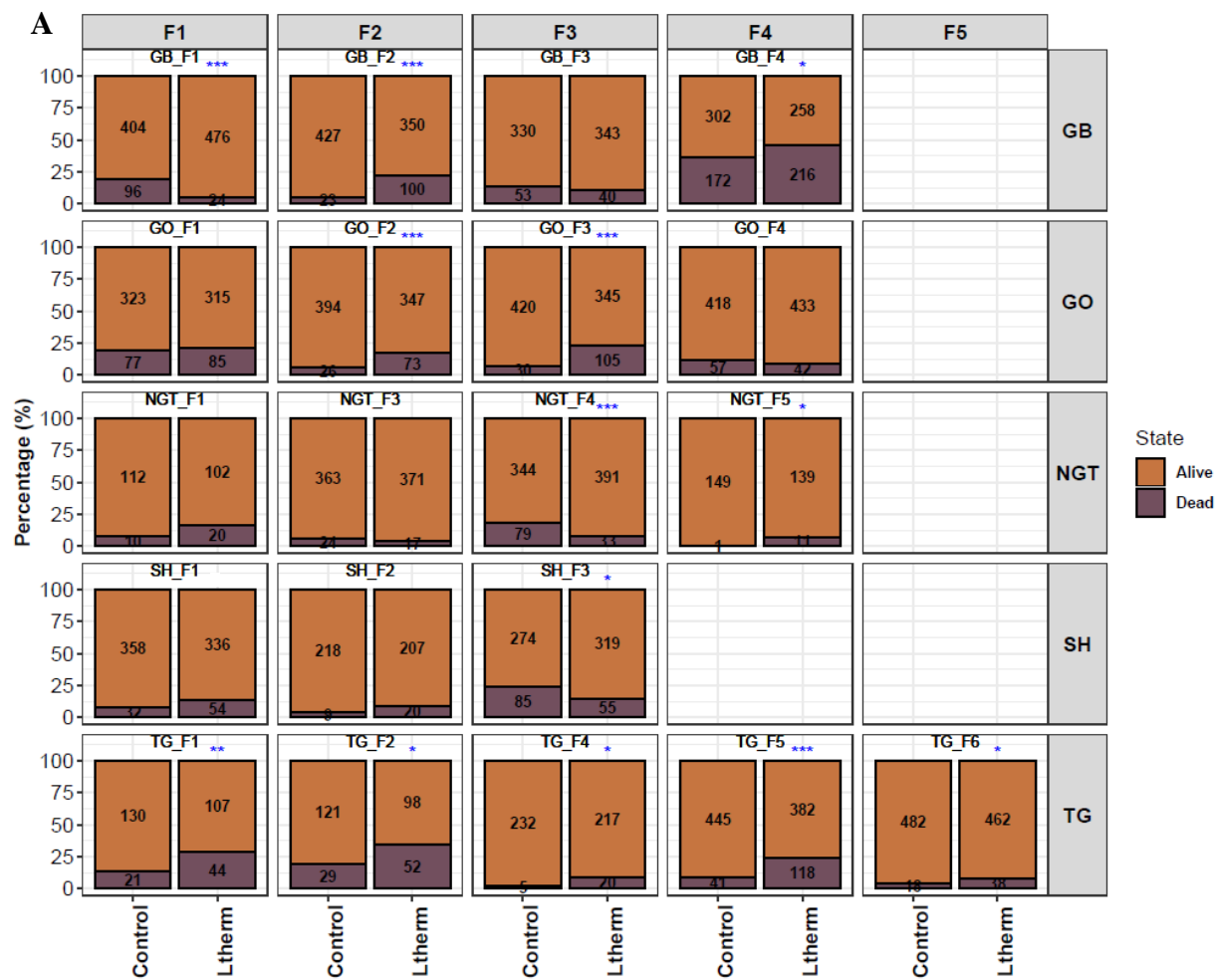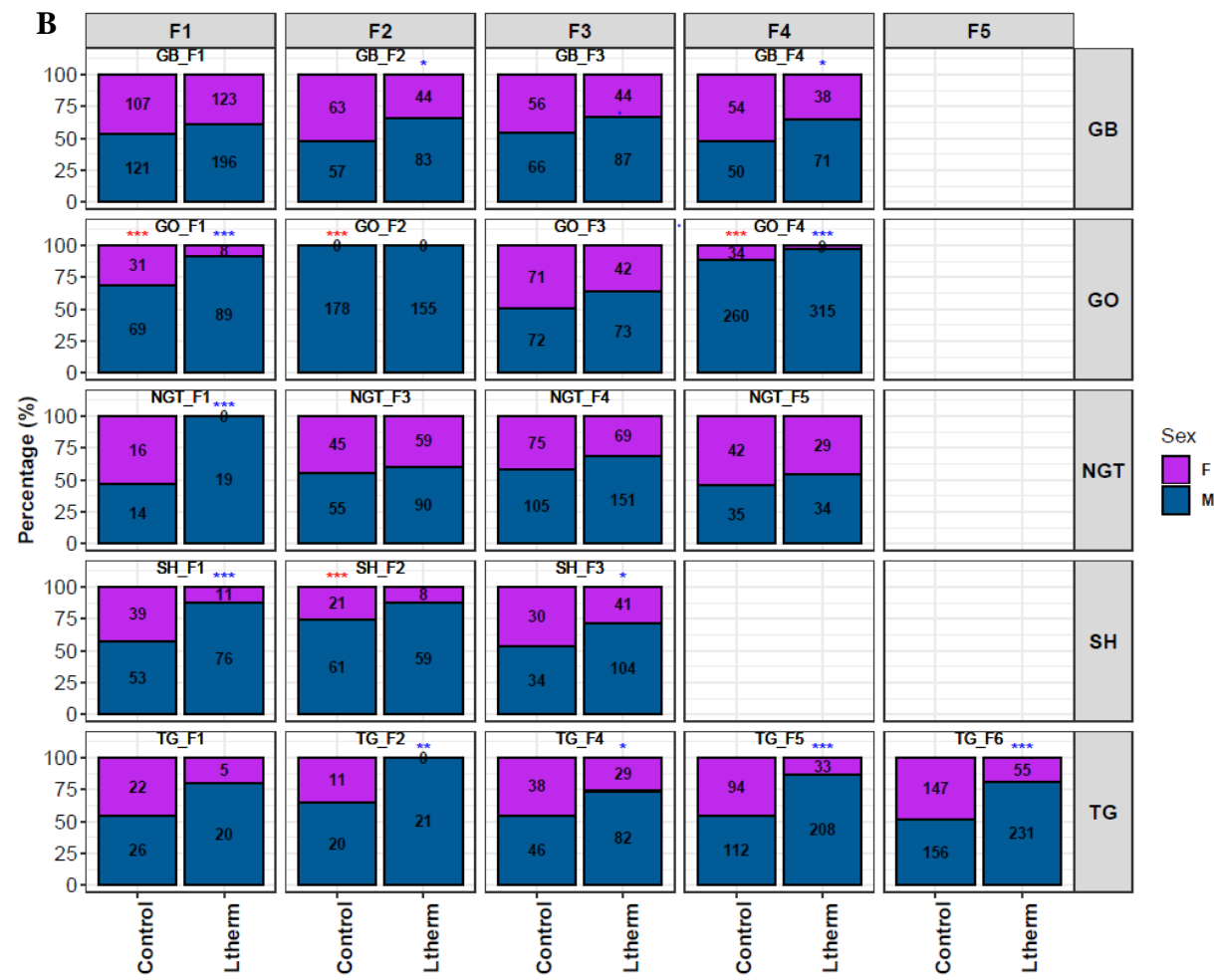

Supplement: dvad009_Supp [file dvad009_supp.zip › suppl_data/Supplementary_Fig 1.pdf]

**A**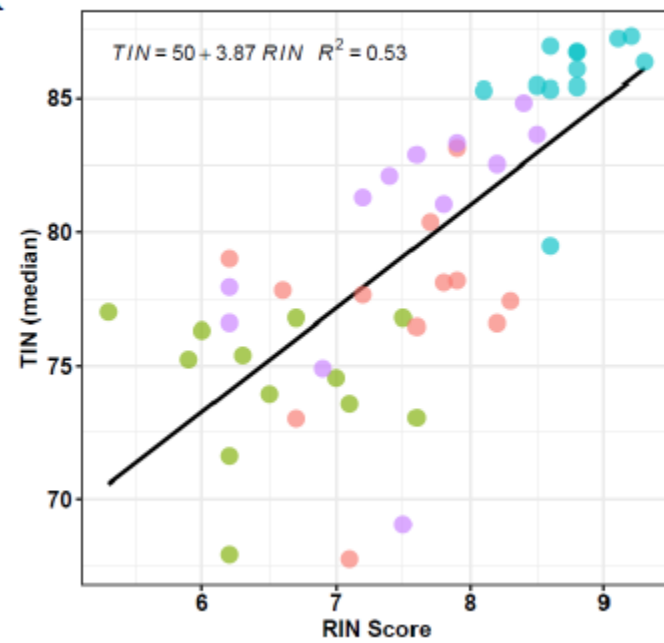**B**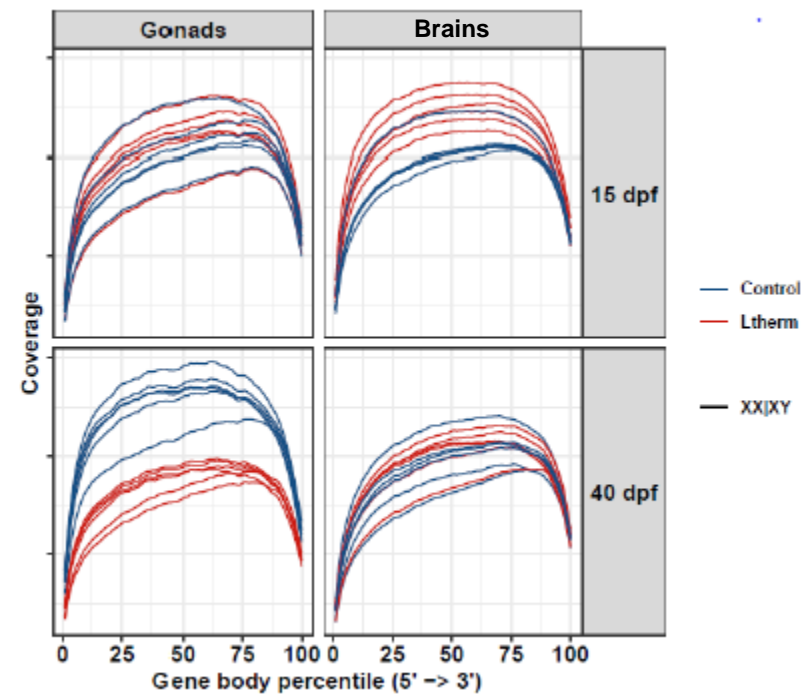

Supplement: dvad009_Supp [file dvad009_supp.zip › suppl_data/Supplementary_Fig 2.pdf]

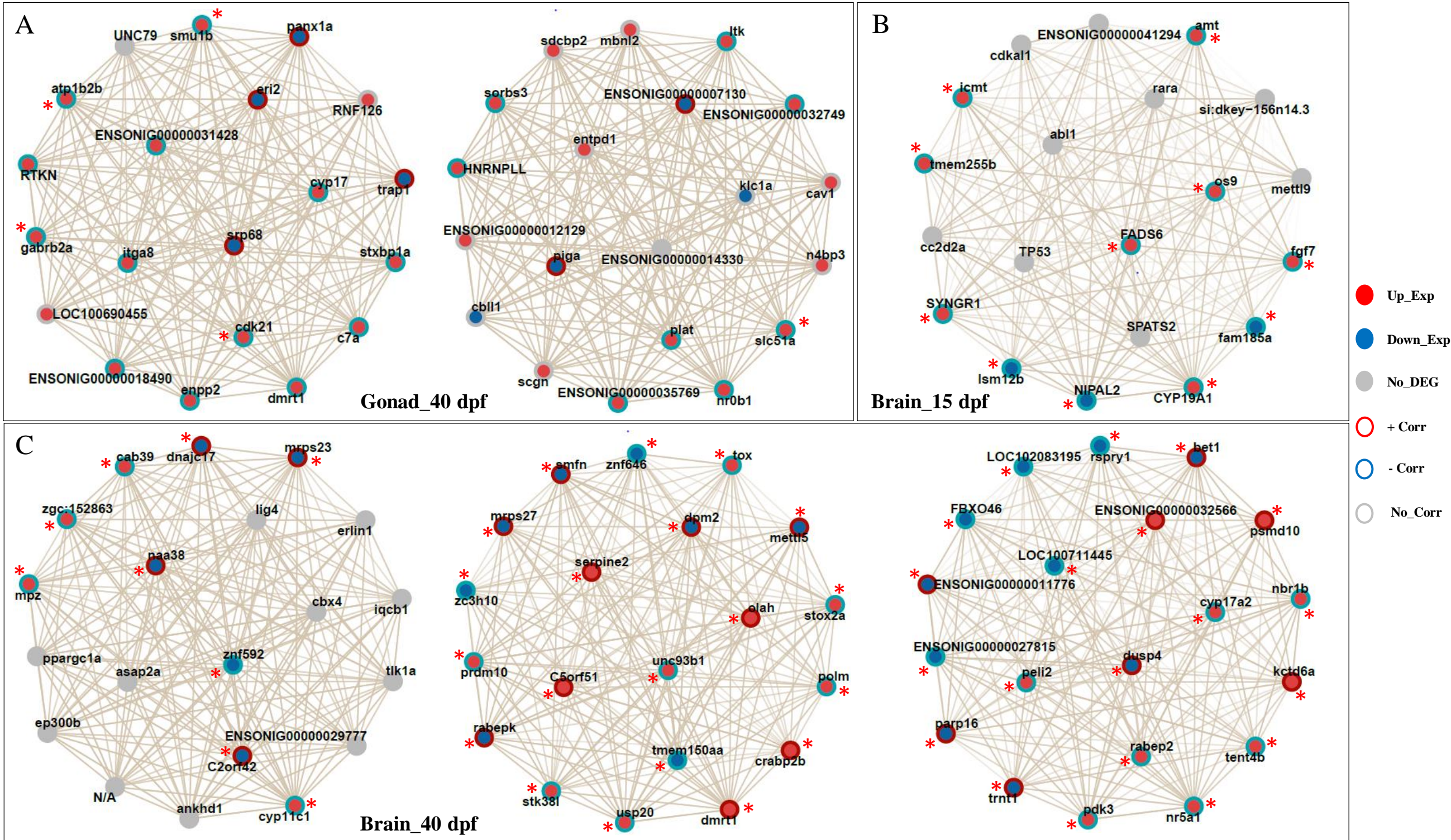

Supplement: dvad009_Supp [file dvad009_supp.zip › suppl_data/Supplementary_Fig 3.pdf]

## Legends

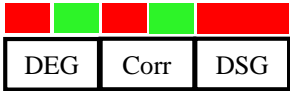

Up\_DEG or +Corr

Down\_DEG or -Corr

B

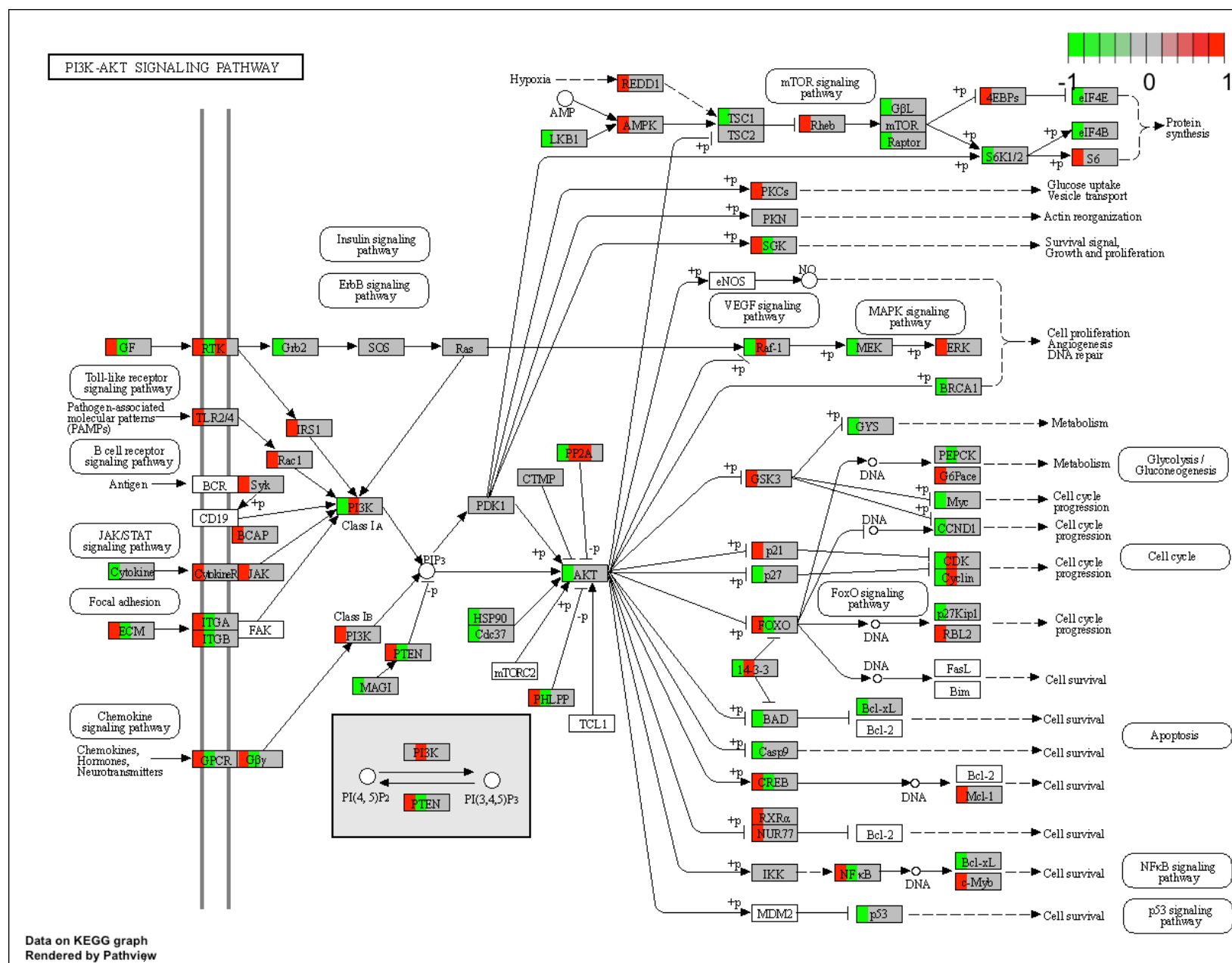

### Legends

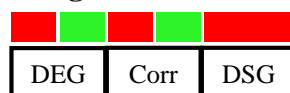

Up\_DEG or +Corr  
Down\_DEG or -Corr

Supplement: dvad009_Supp [file dvad009_supp.zip › suppl_data/Supplementary_Fig 4.pdf]

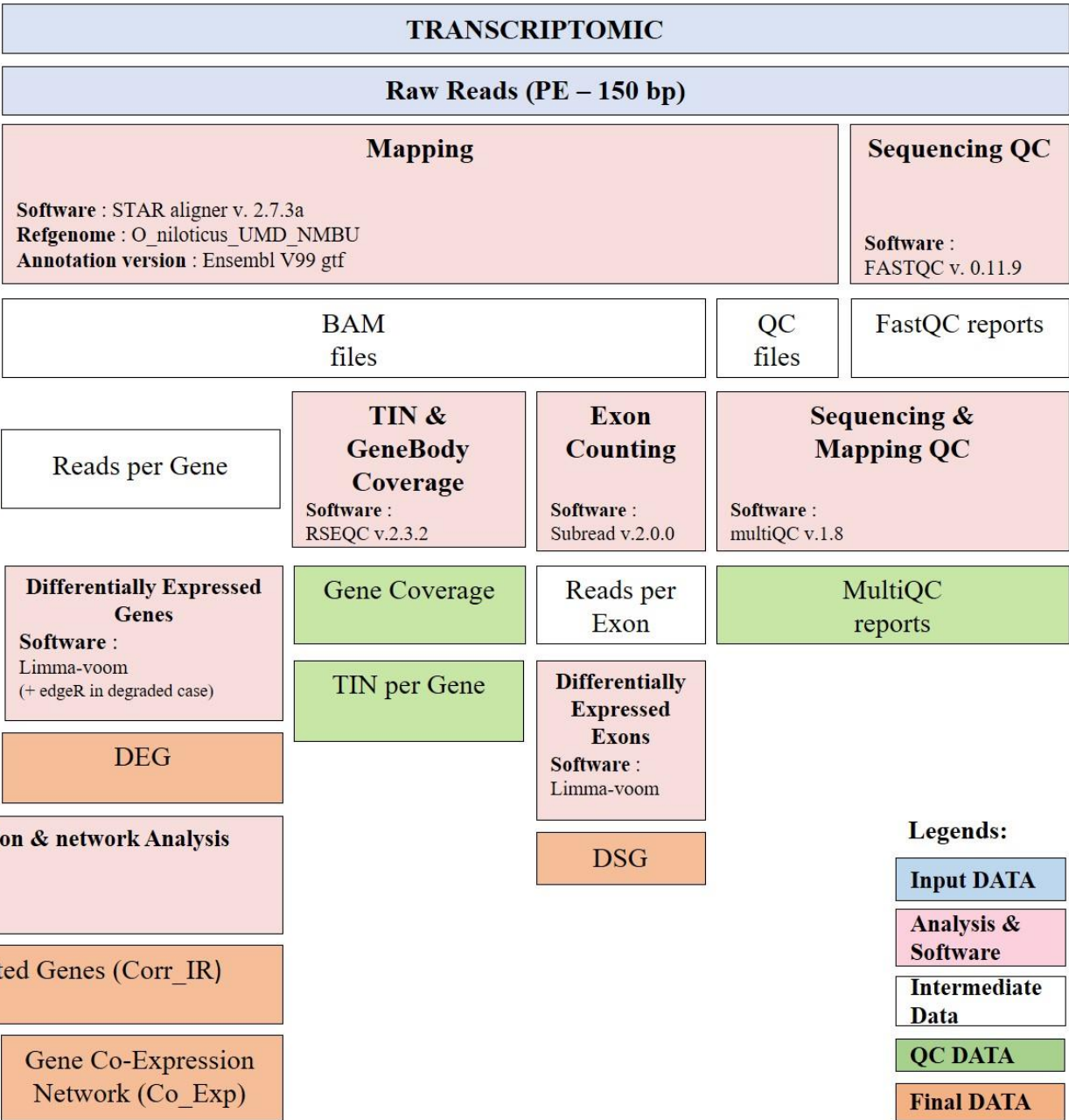

Supplement: dvad009_Supp [file dvad009_supp.zip › suppl_data/Supplementary_Fig 5.pdf]
